# Supplementary figures and images for: Multi-Omics Unveils Inflammatory Regulation of Fermented Sini Decoction Dregs in Broilers Infected with Avian Pathogenic Escherichia coli
Source: Vet Sci. 2025 May 15;12(5):479. doi: 10.3390/vetsci12050479 (PMC12116022; doi:10.3390/vetsci12050479)

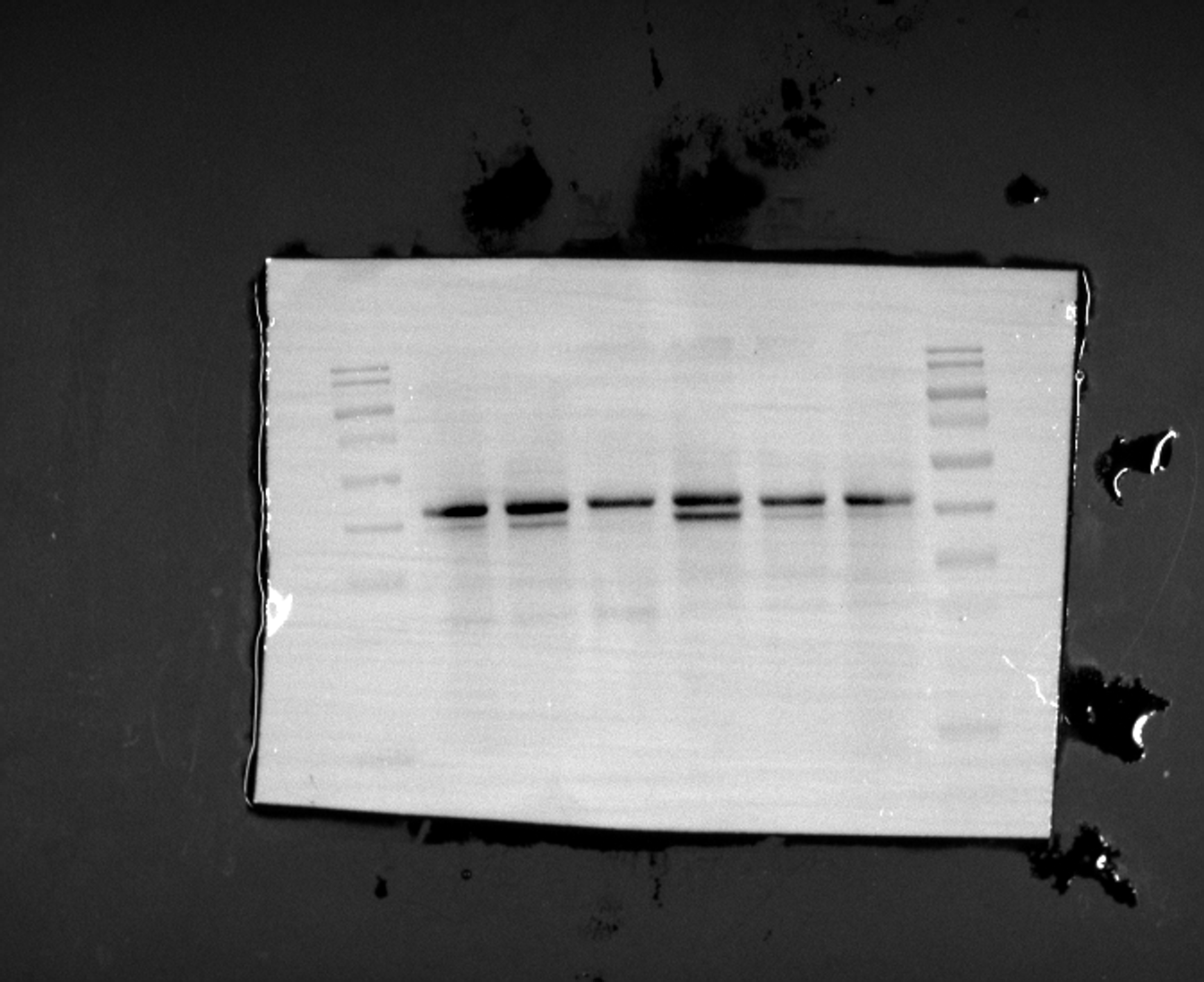

Supplement: Supplementary file 1 [file vetsci-12-00479-s001.zip › Western-blot/cell/细胞jun-pjun-actin/actin.Tif]

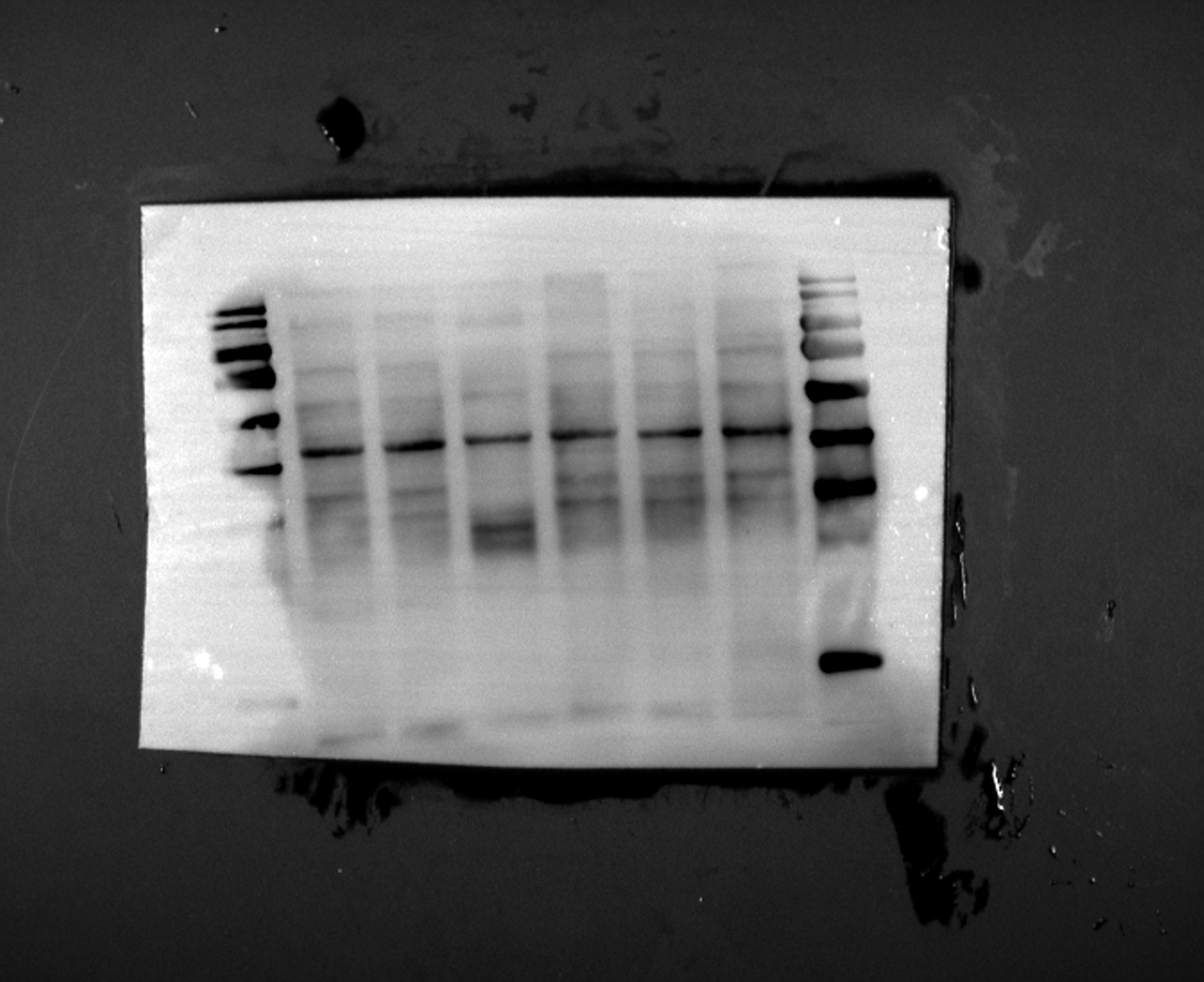

Supplement: Supplementary file 1 [file vetsci-12-00479-s001.zip › Western-blot/cell/细胞jun-pjun-actin/JUN.Tif]

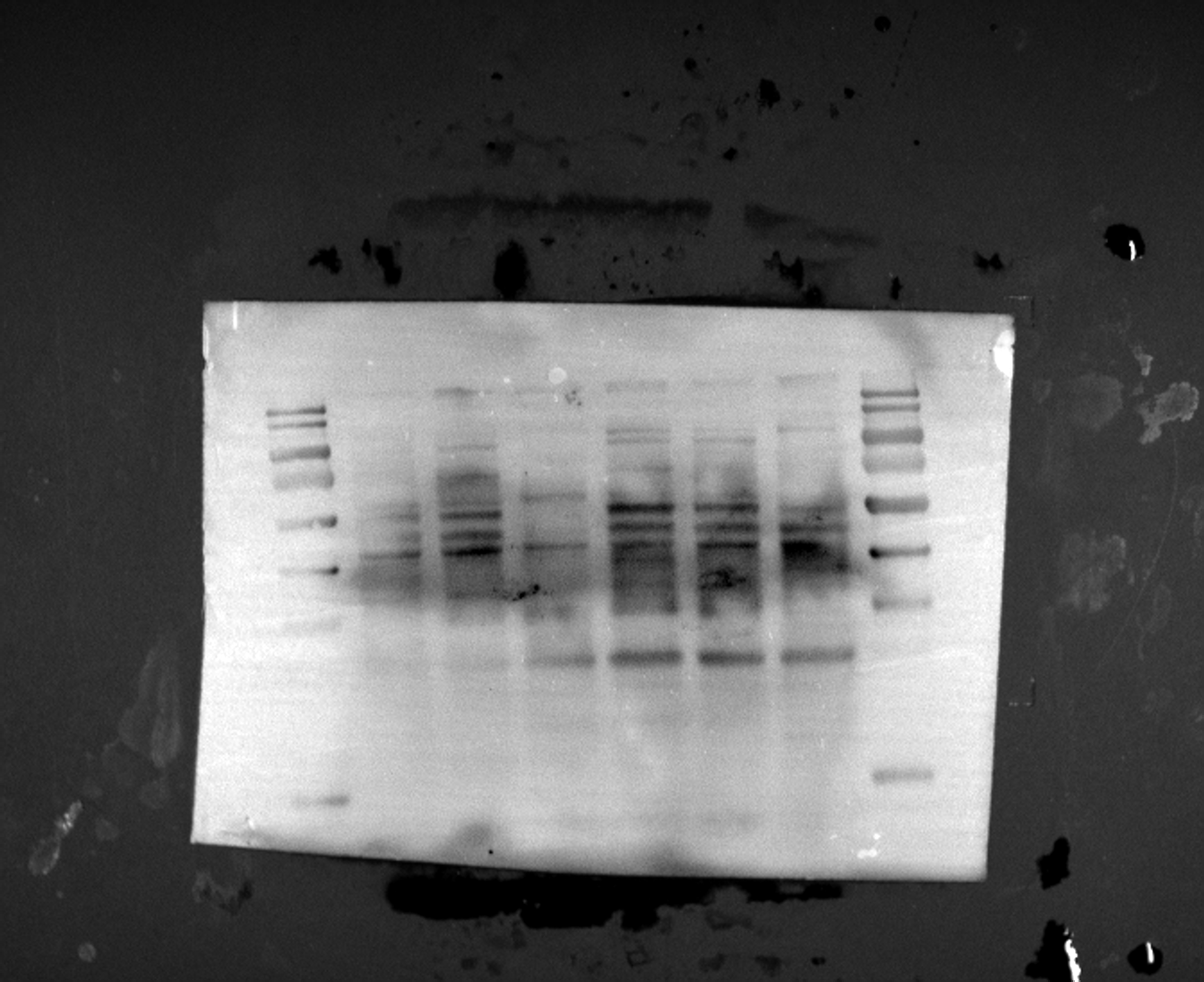

Supplement: Supplementary file 1 [file vetsci-12-00479-s001.zip › Western-blot/cell/细胞jun-pjun-actin/p-JUN.Tif]

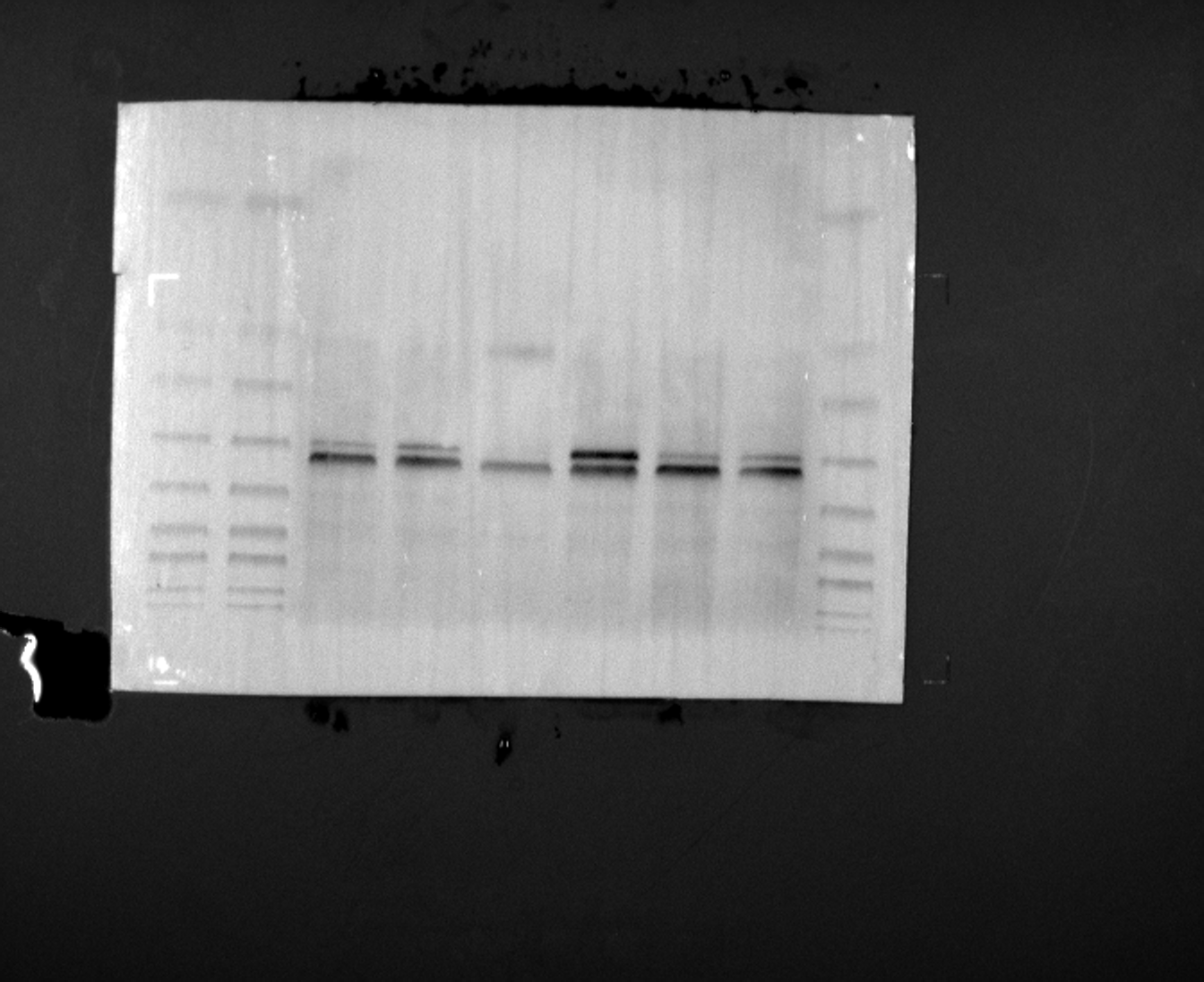

Supplement: Supplementary file 1 [file vetsci-12-00479-s001.zip › Western-blot/cell/细胞myd88/actin.Tif]

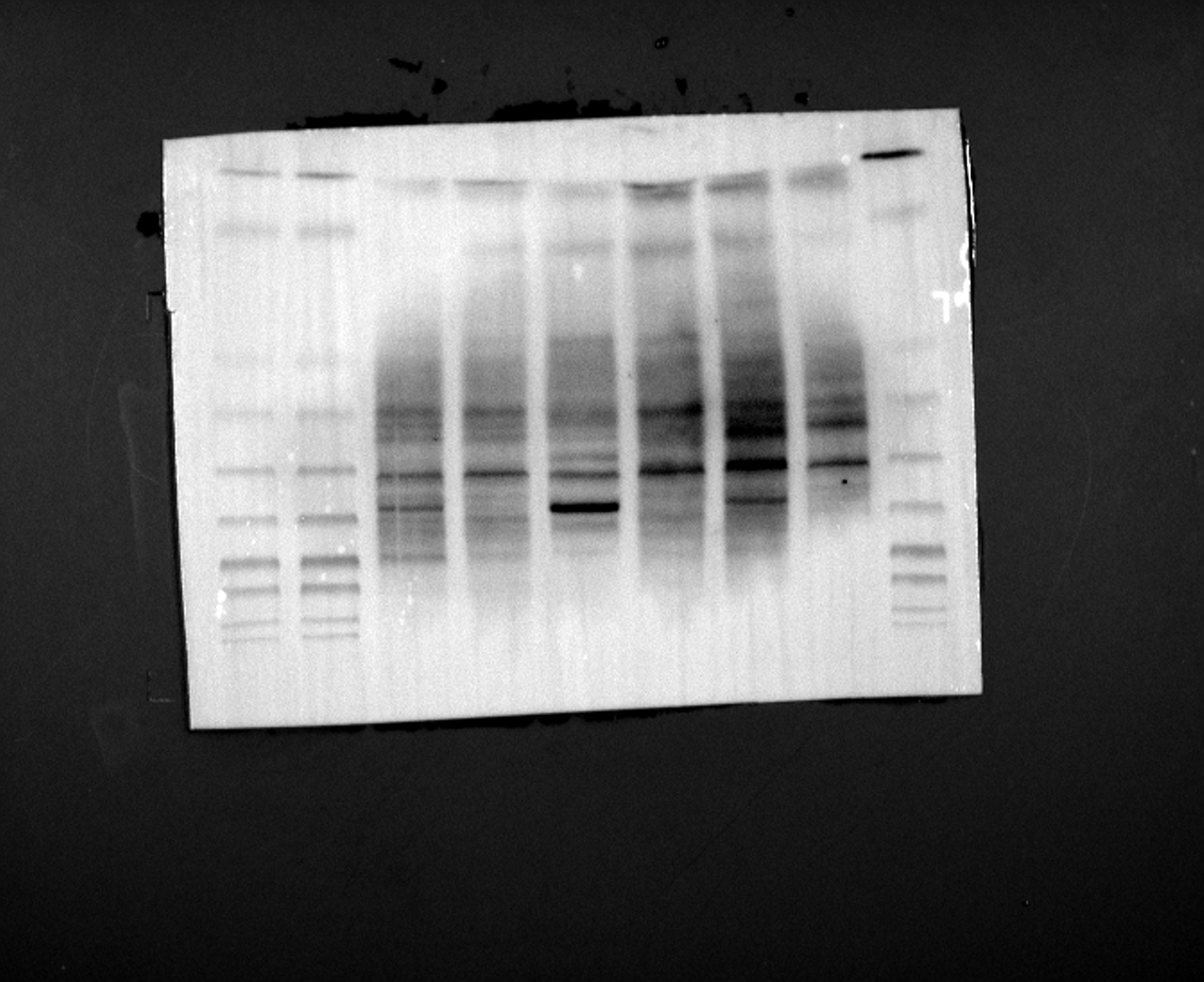

Supplement: Supplementary file 1 [file vetsci-12-00479-s001.zip › Western-blot/cell/细胞myd88/myd88.Tif]

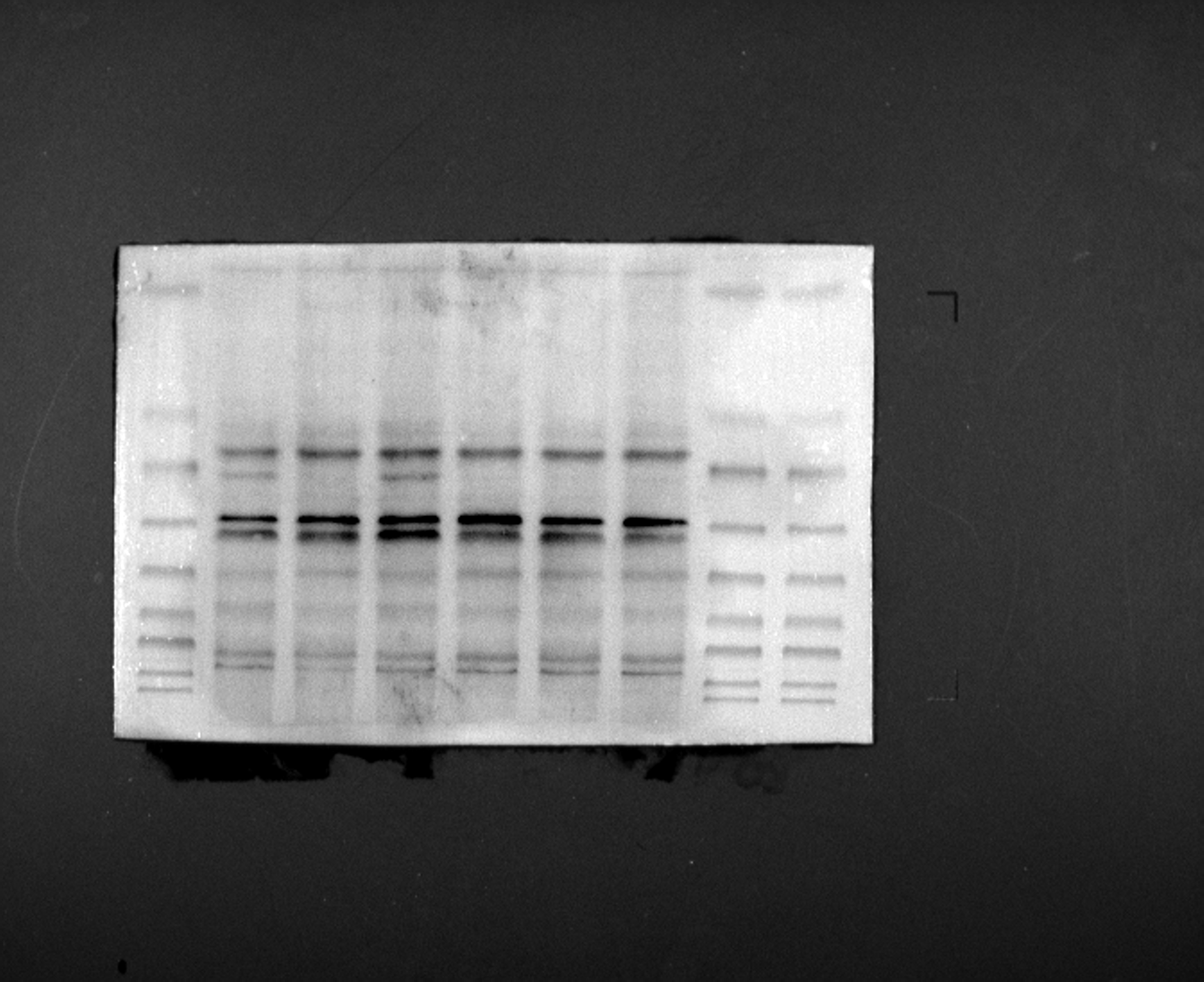

Supplement: Supplementary file 1 [file vetsci-12-00479-s001.zip › Western-blot/cell/细胞pikb-ikb-actin/actin.Tif]

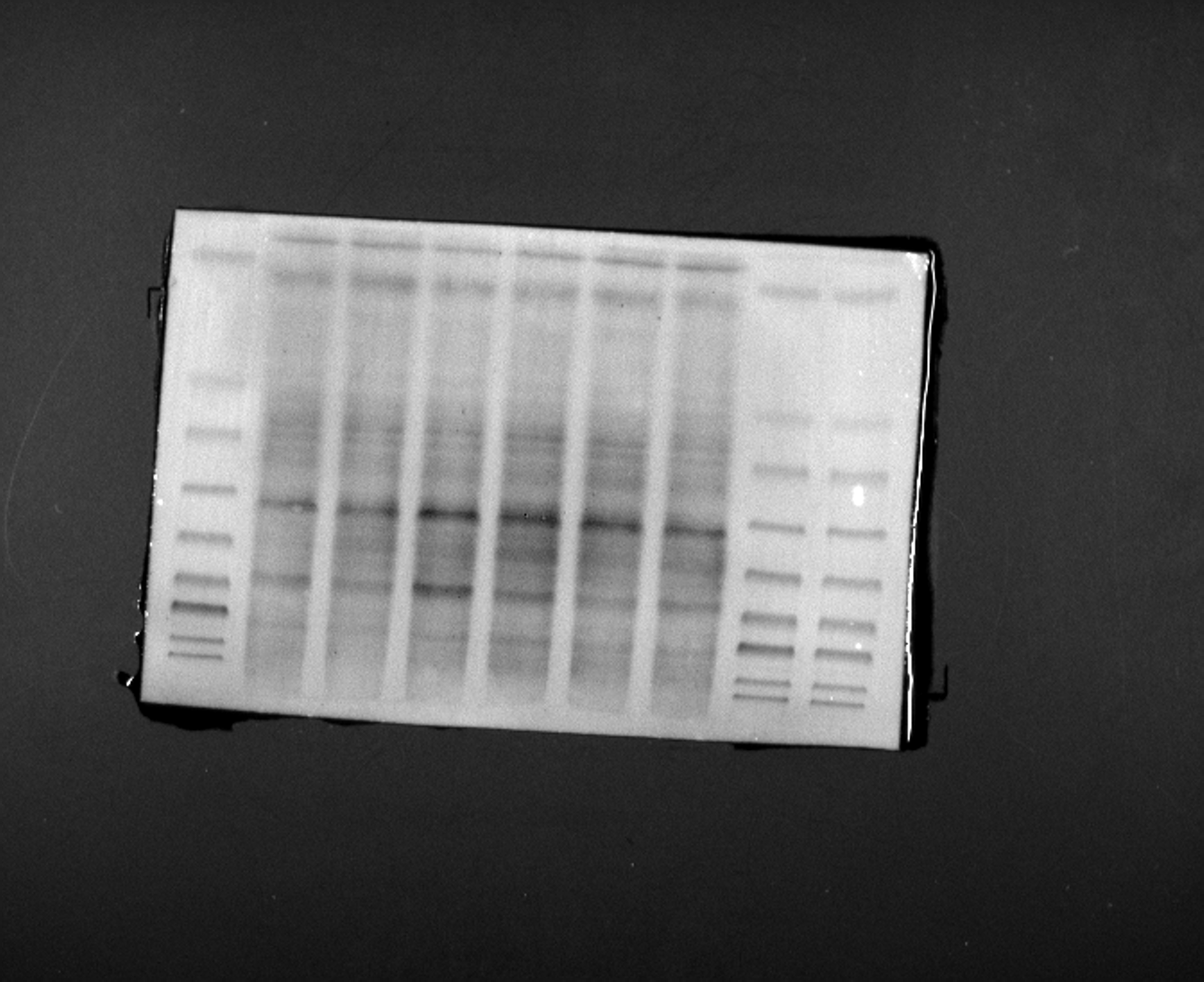

Supplement: Supplementary file 1 [file vetsci-12-00479-s001.zip › Western-blot/cell/细胞pikb-ikb-actin/ikb.Tif]

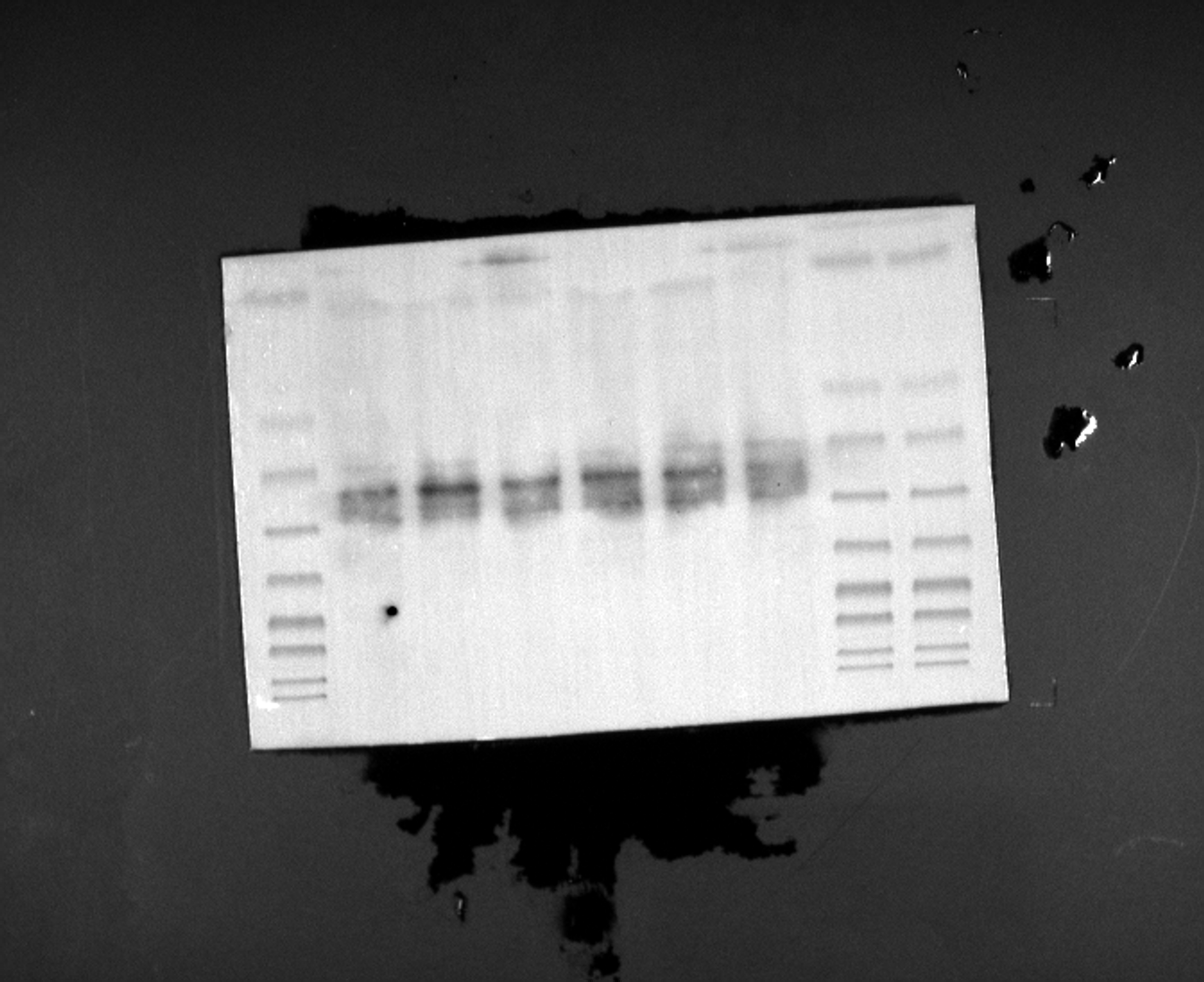

Supplement: Supplementary file 1 [file vetsci-12-00479-s001.zip › Western-blot/cell/细胞pikb-ikb-actin/PIKB.Tif]

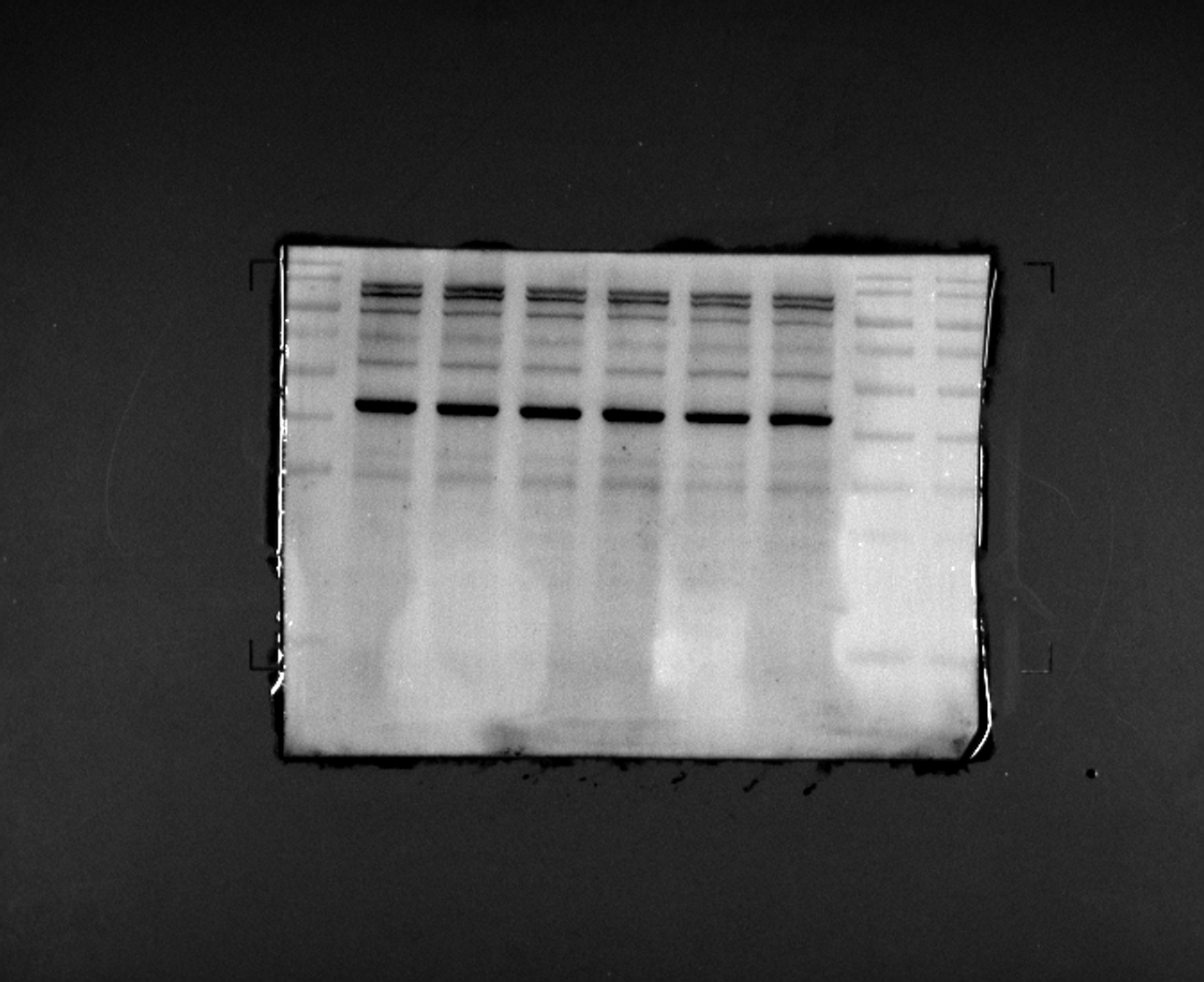

Supplement: Supplementary file 1 [file vetsci-12-00479-s001.zip › Western-blot/cell/细胞pp38-p38-actin/actin.Tif]

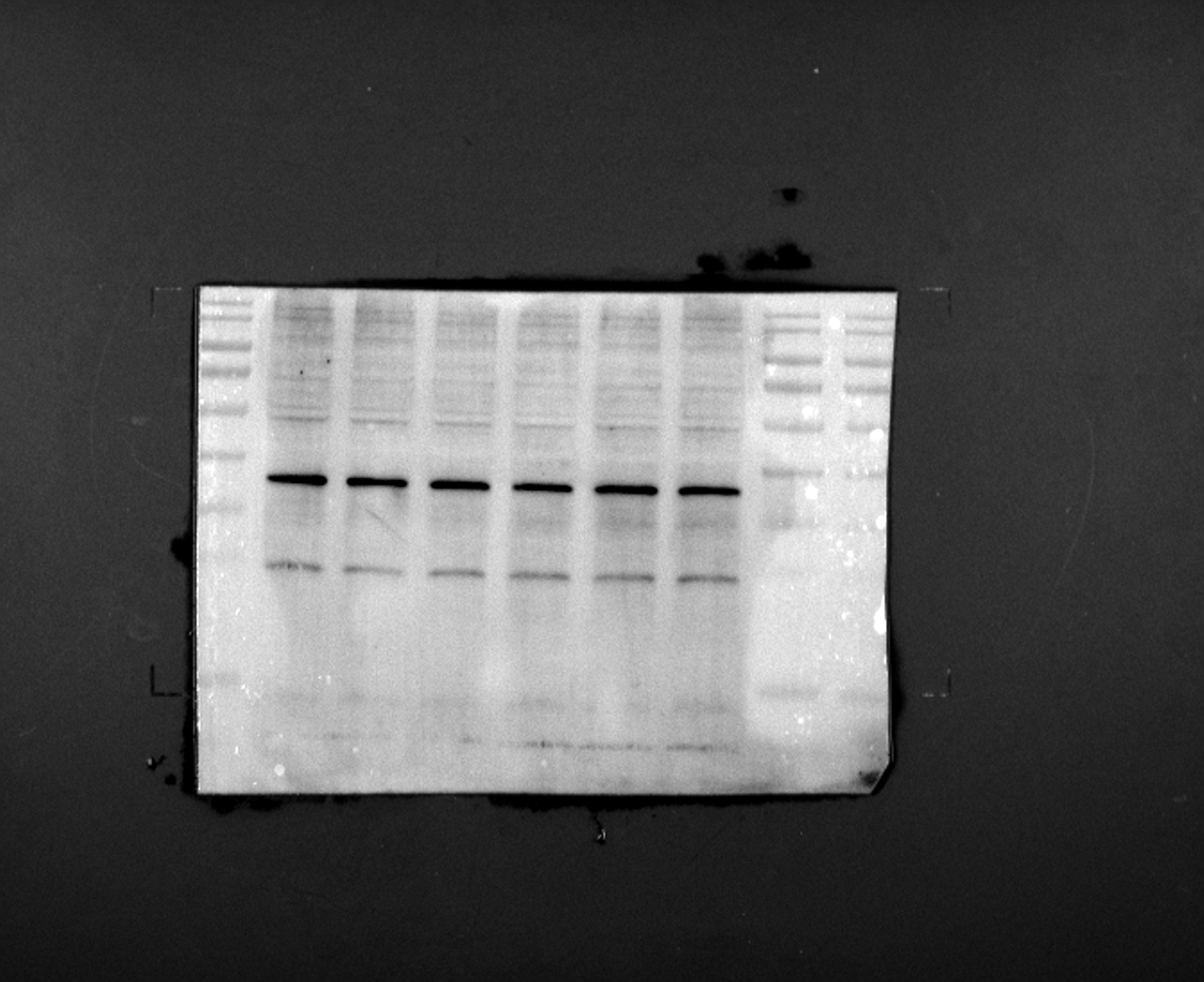

Supplement: Supplementary file 1 [file vetsci-12-00479-s001.zip › Western-blot/cell/细胞pp38-p38-actin/p38.Tif]

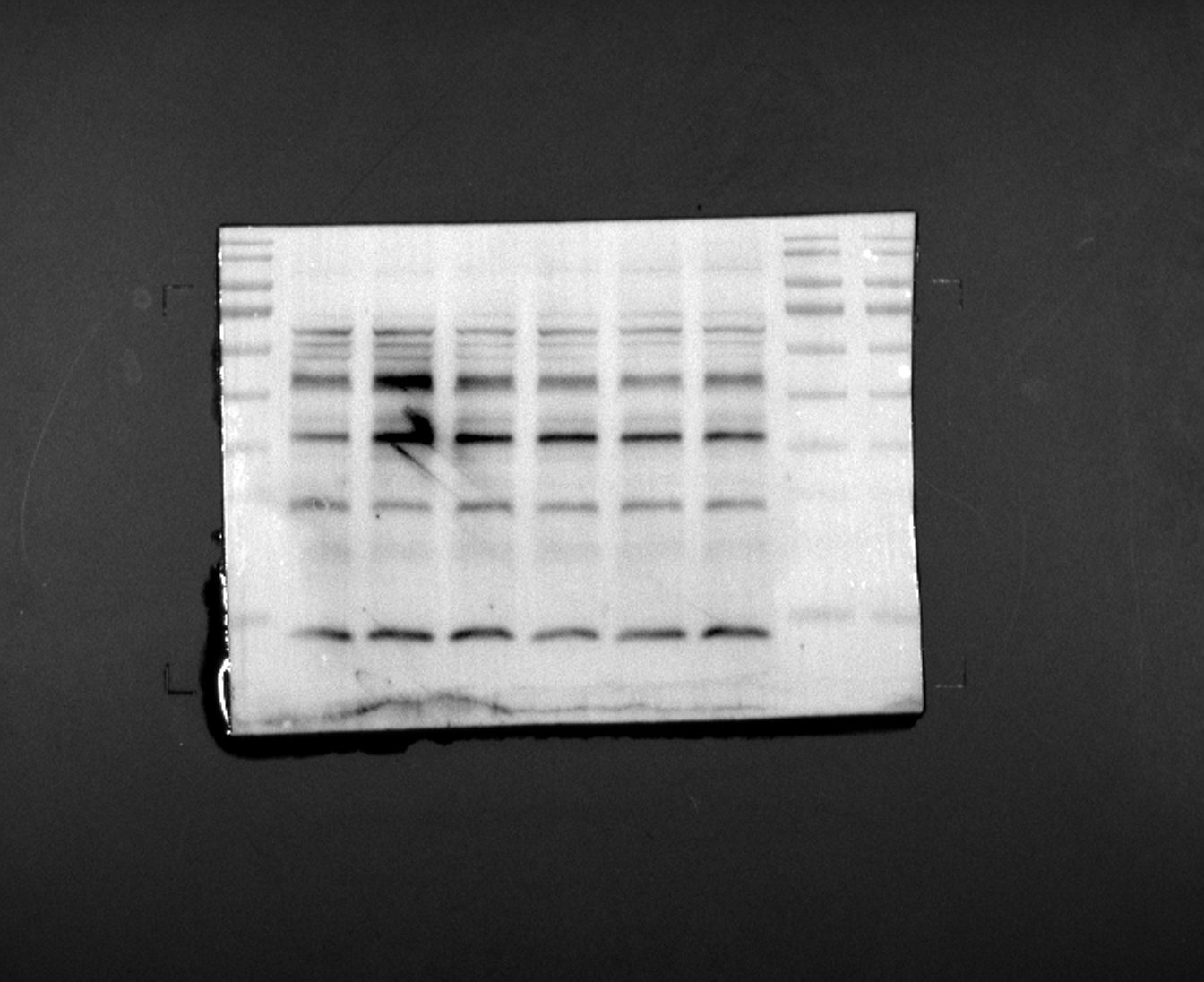

Supplement: Supplementary file 1 [file vetsci-12-00479-s001.zip › Western-blot/cell/细胞pp38-p38-actin/pp38.Tif]

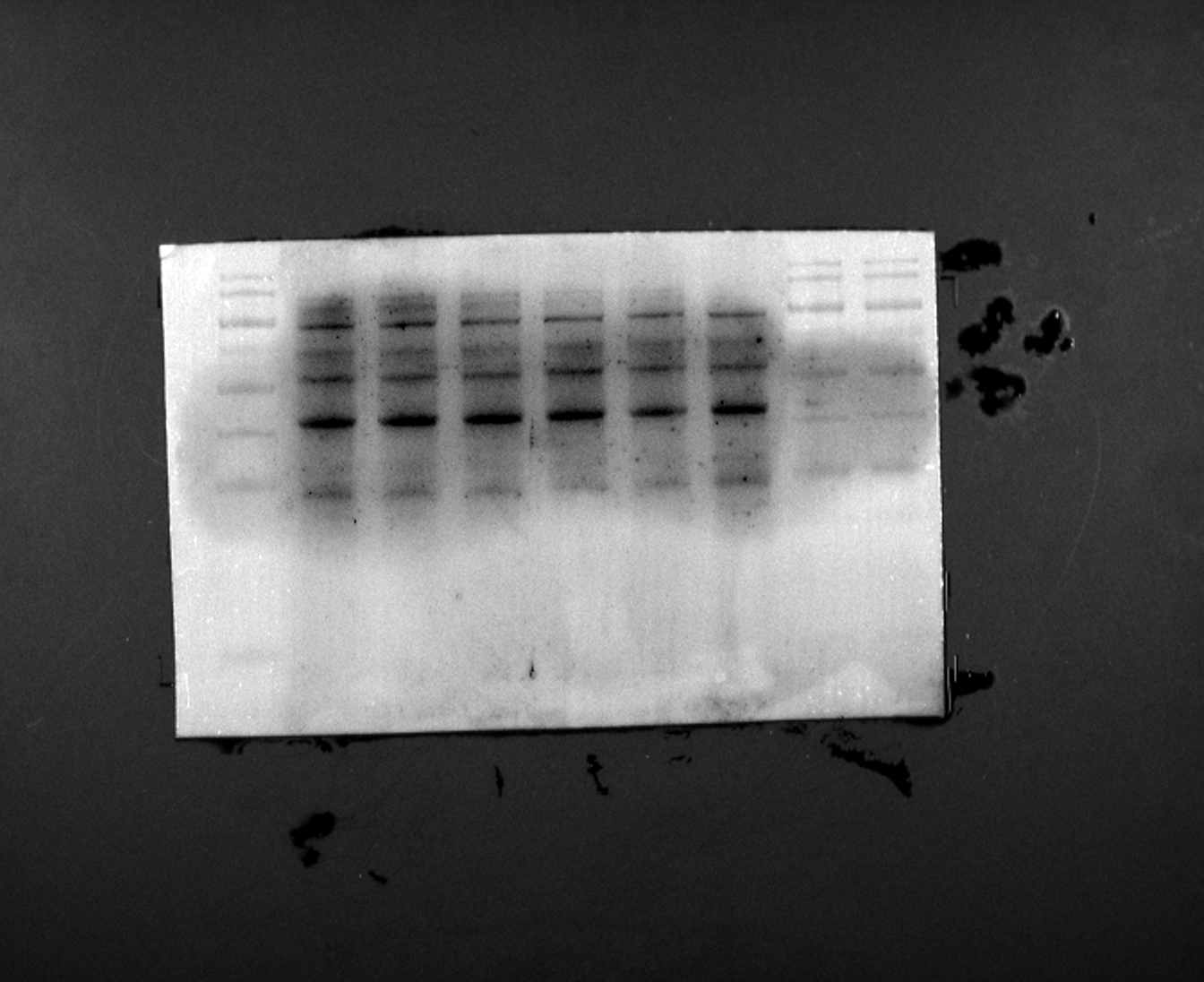

Supplement: Supplementary file 1 [file vetsci-12-00479-s001.zip › Western-blot/cell/细胞pp65-p65-actin/actin.Tif]

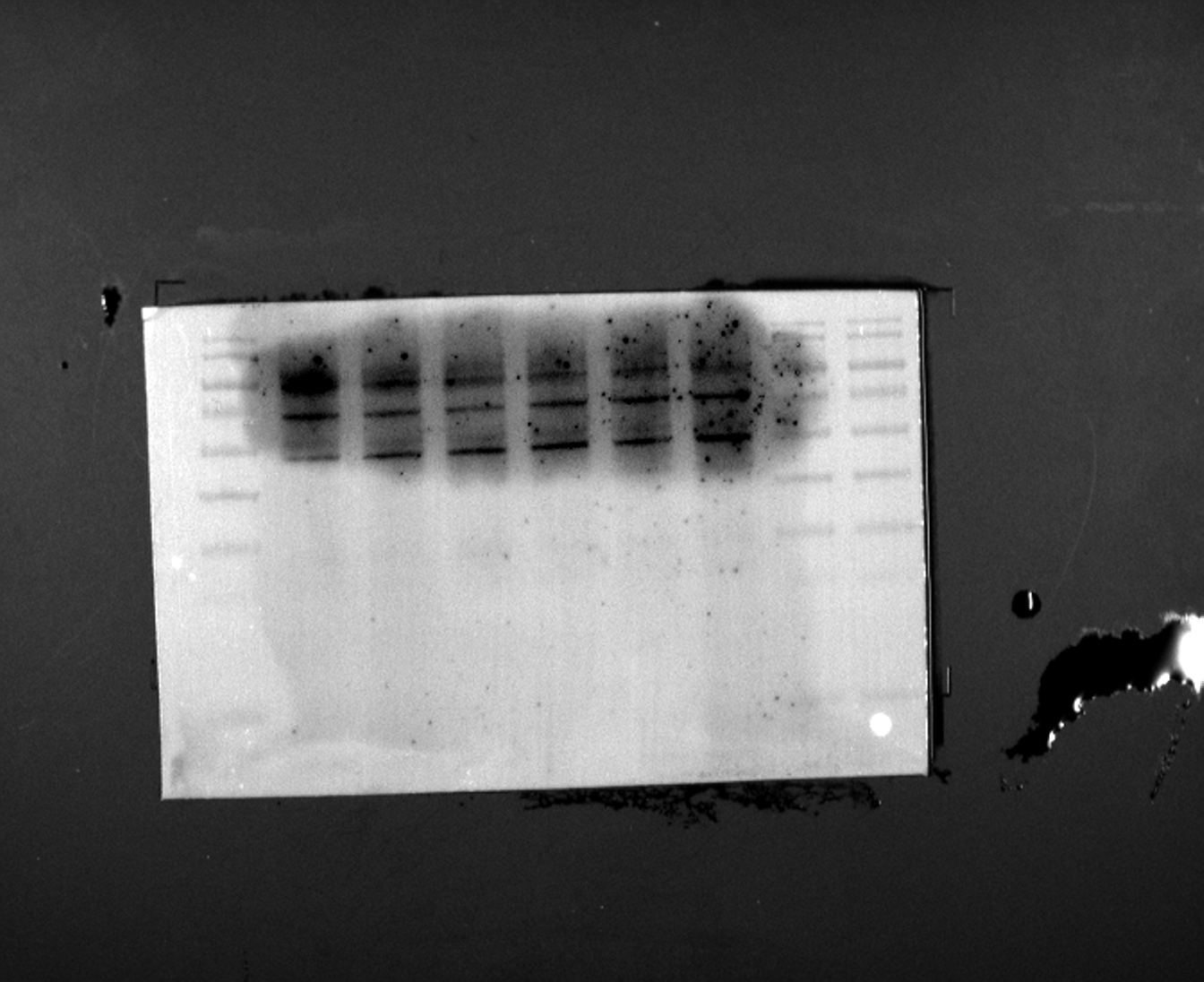

Supplement: Supplementary file 1 [file vetsci-12-00479-s001.zip › Western-blot/cell/细胞pp65-p65-actin/p65.Tif]

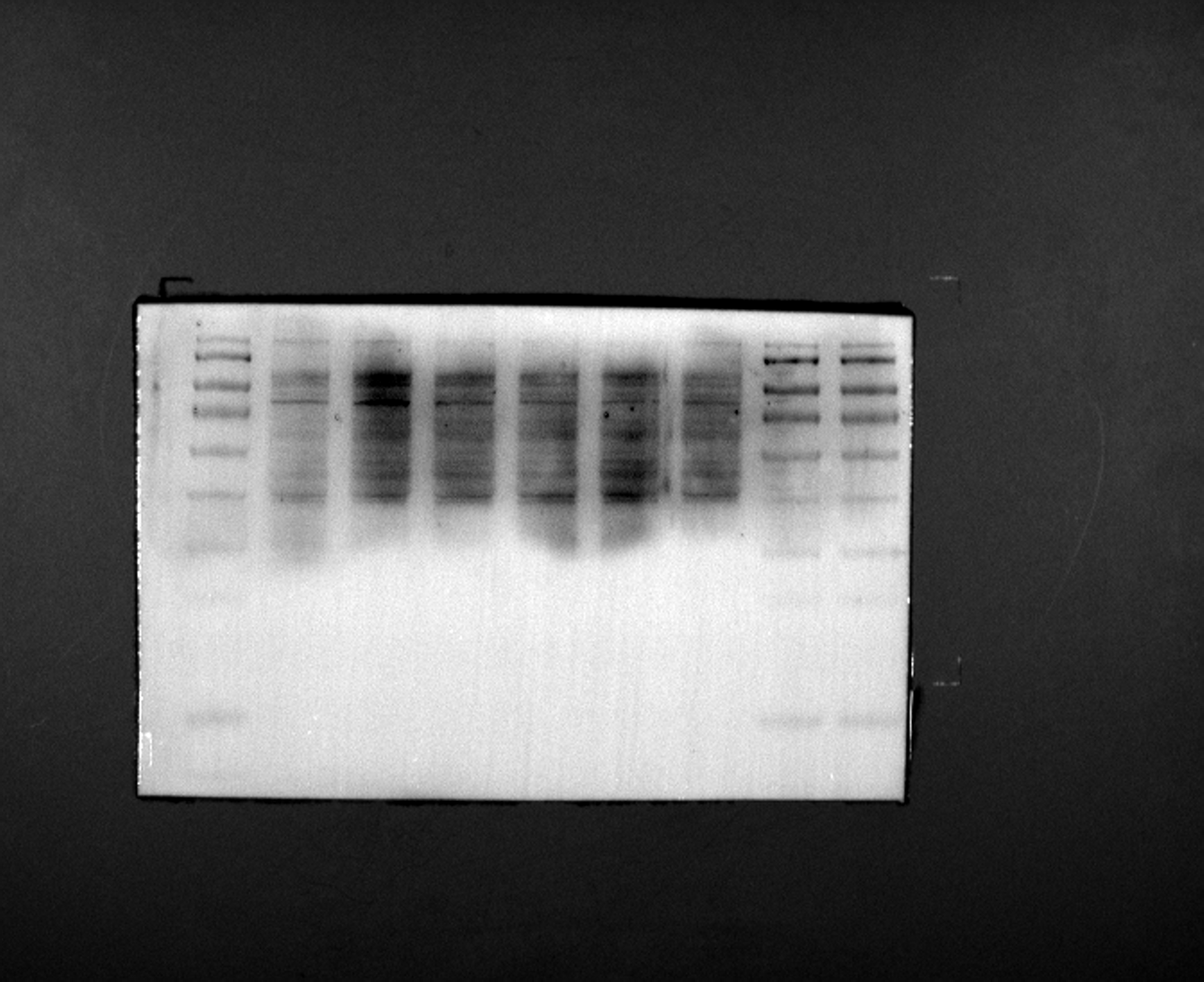

Supplement: Supplementary file 1 [file vetsci-12-00479-s001.zip › Western-blot/cell/细胞pp65-p65-actin/pp65.Tif]

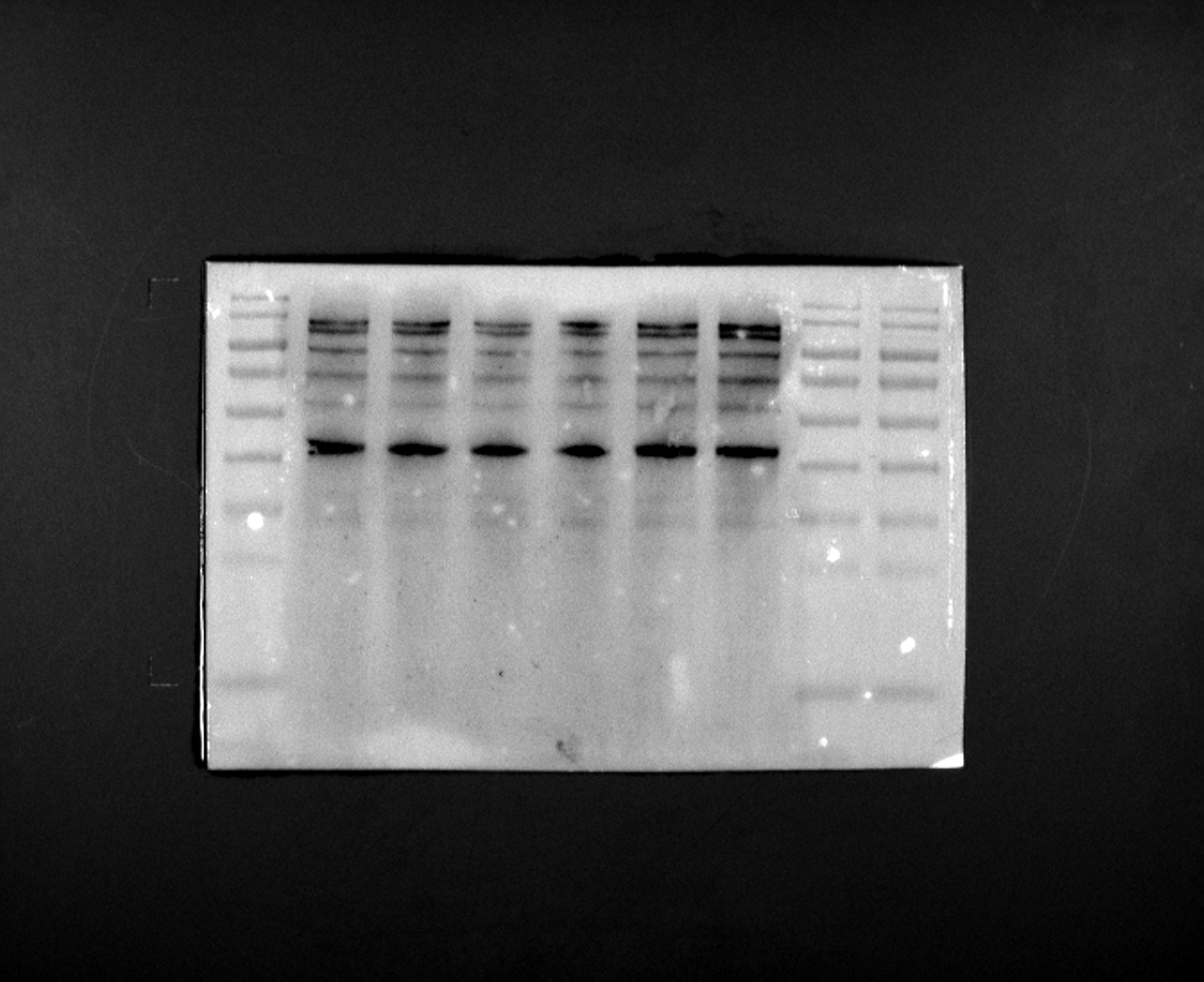

Supplement: Supplementary file 1 [file vetsci-12-00479-s001.zip › Western-blot/cell/细胞tlr4/actin.Tif]

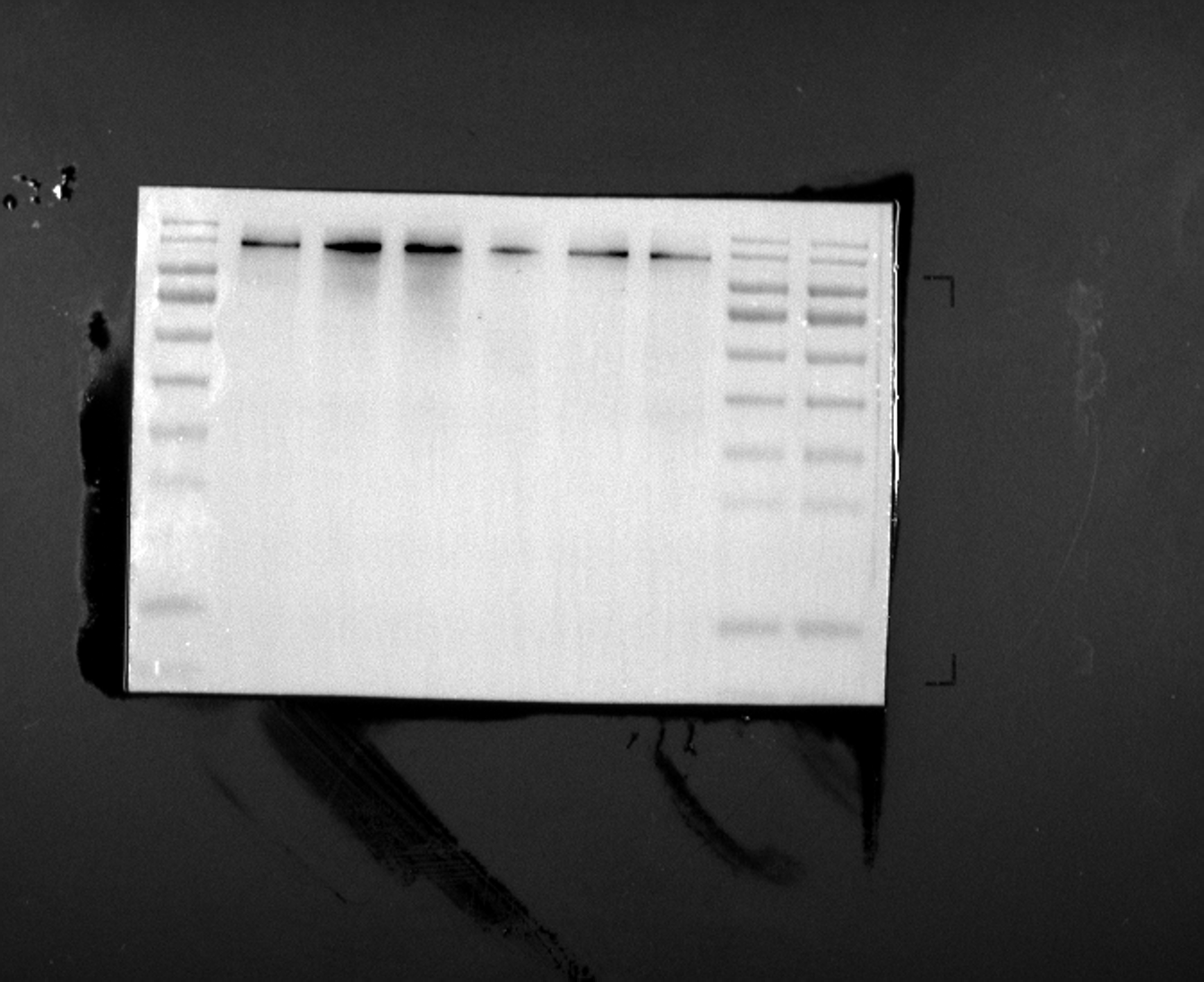

Supplement: Supplementary file 1 [file vetsci-12-00479-s001.zip › Western-blot/cell/细胞tlr4/tlr4.Tif]

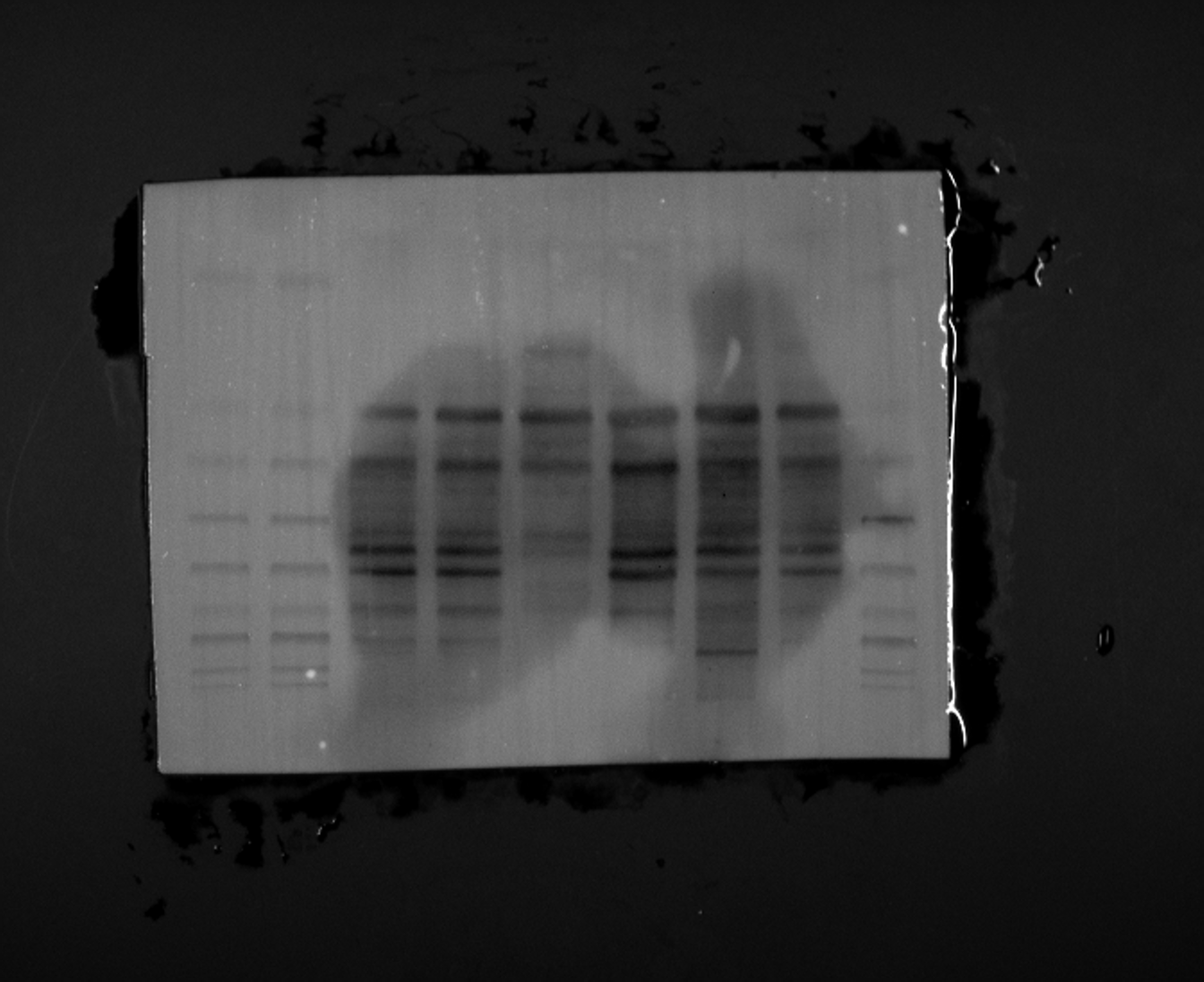

Supplement: Supplementary file 1 [file vetsci-12-00479-s001.zip › Western-blot/chicken/组织jun-pjun-actin/JUN.Tif]

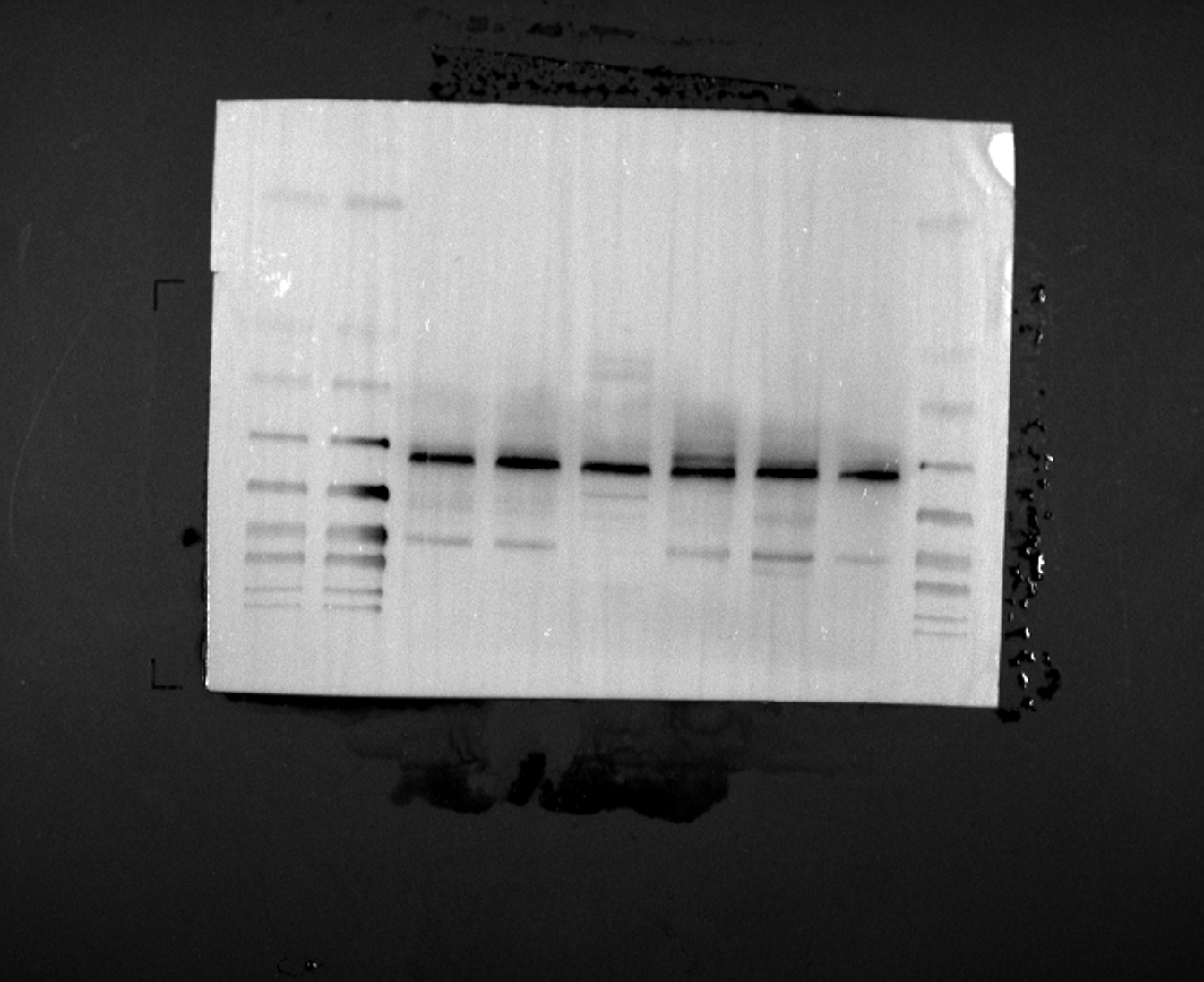

Supplement: Supplementary file 1 [file vetsci-12-00479-s001.zip › Western-blot/chicken/组织jun-pjun-actin/pJUN.Tif]

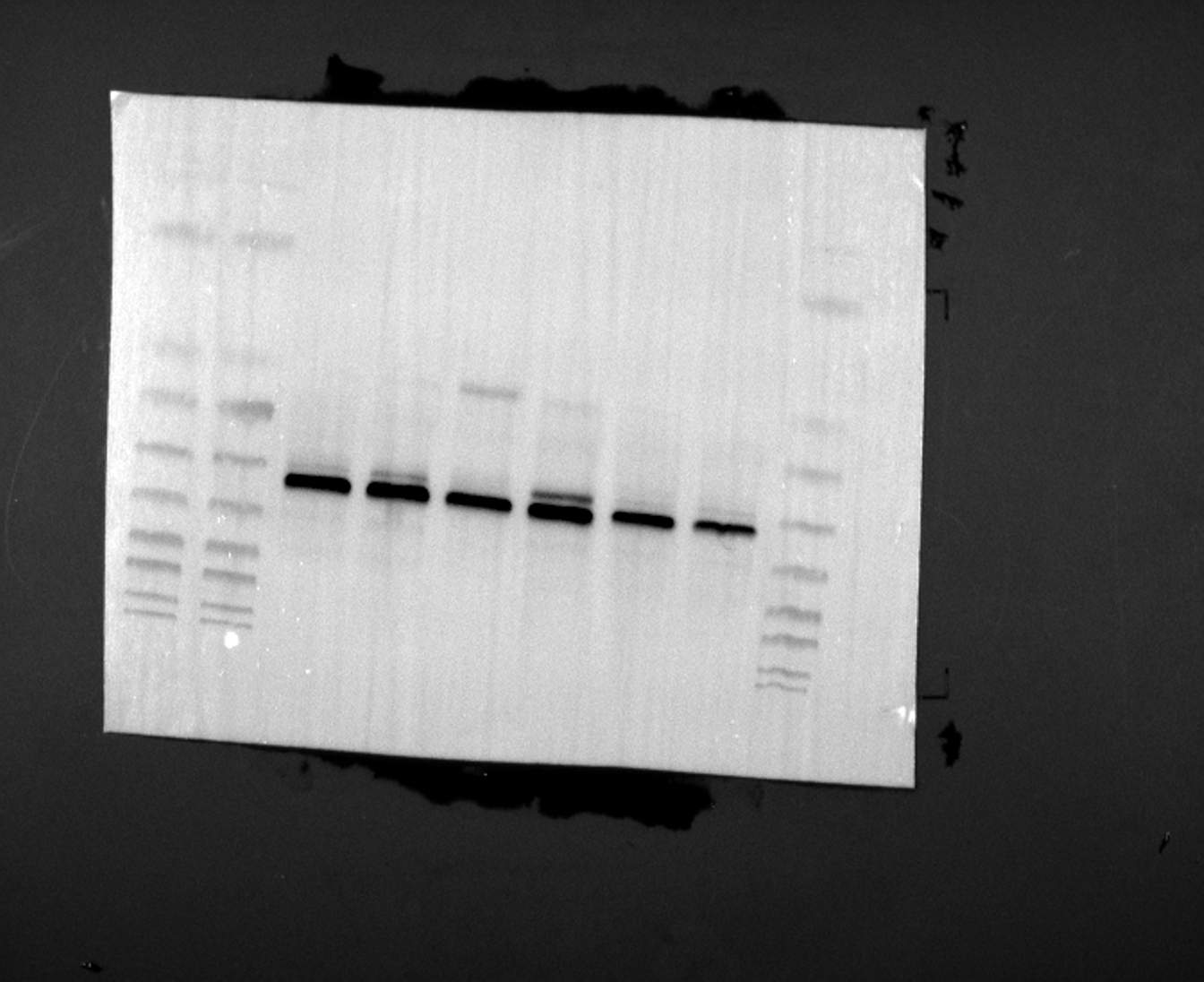

Supplement: Supplementary file 1 [file vetsci-12-00479-s001.zip › Western-blot/chicken/组织myd88/actin.Tif]

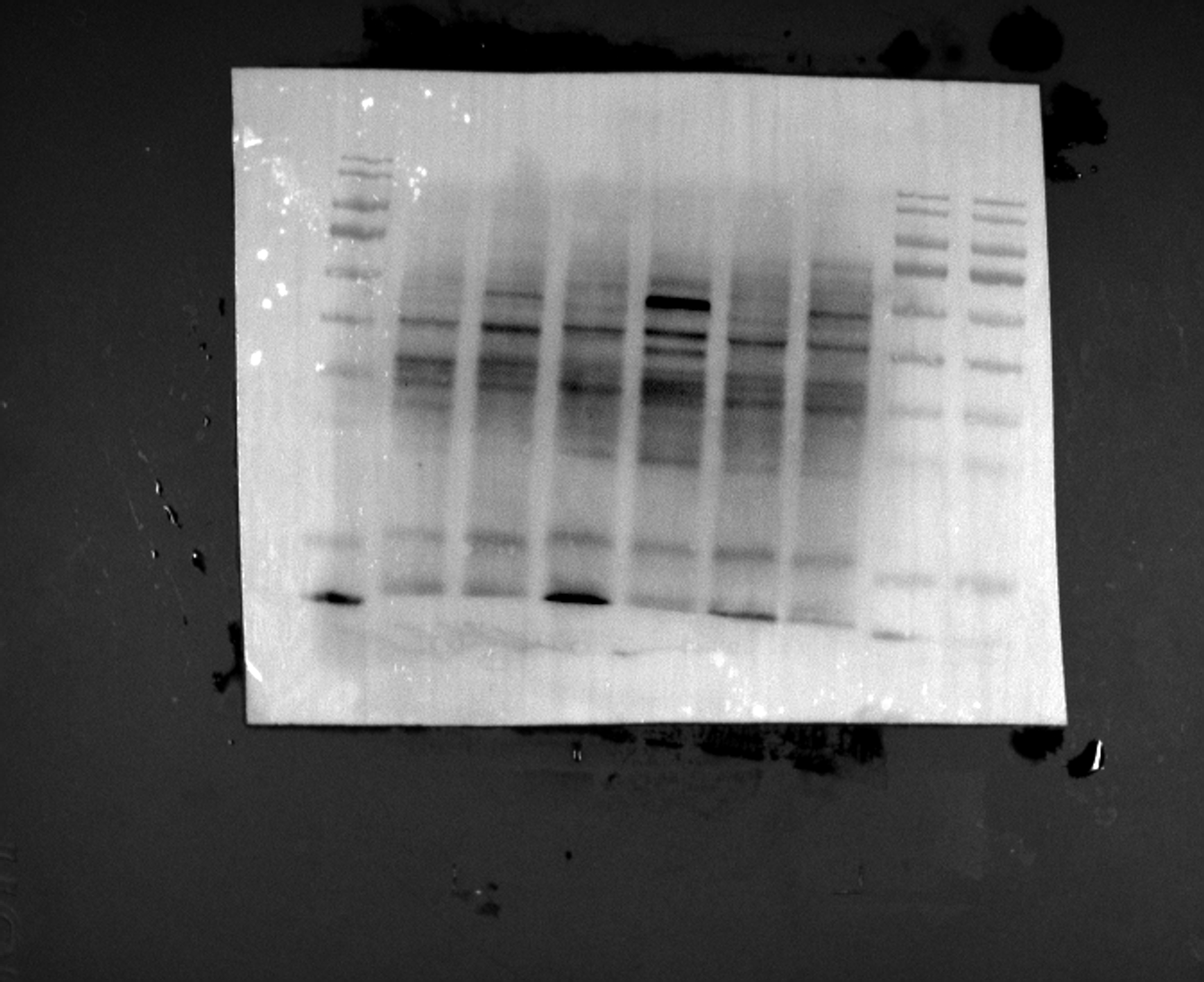

Supplement: Supplementary file 1 [file vetsci-12-00479-s001.zip › Western-blot/chicken/组织myd88/MyD88.Tif]

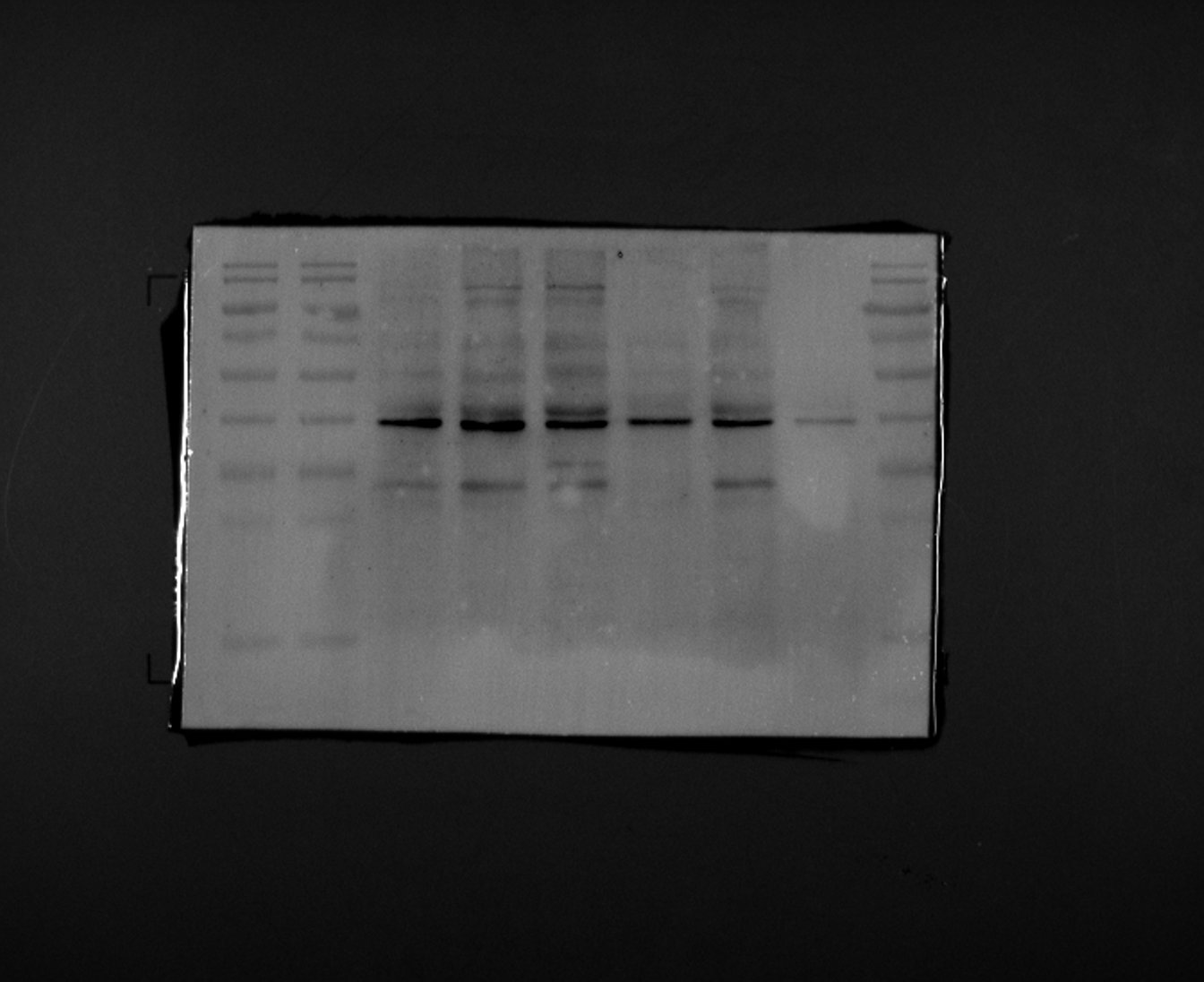

Supplement: Supplementary file 1 [file vetsci-12-00479-s001.zip › Western-blot/chicken/组织pikb-ikb-actin/actin.Tif]

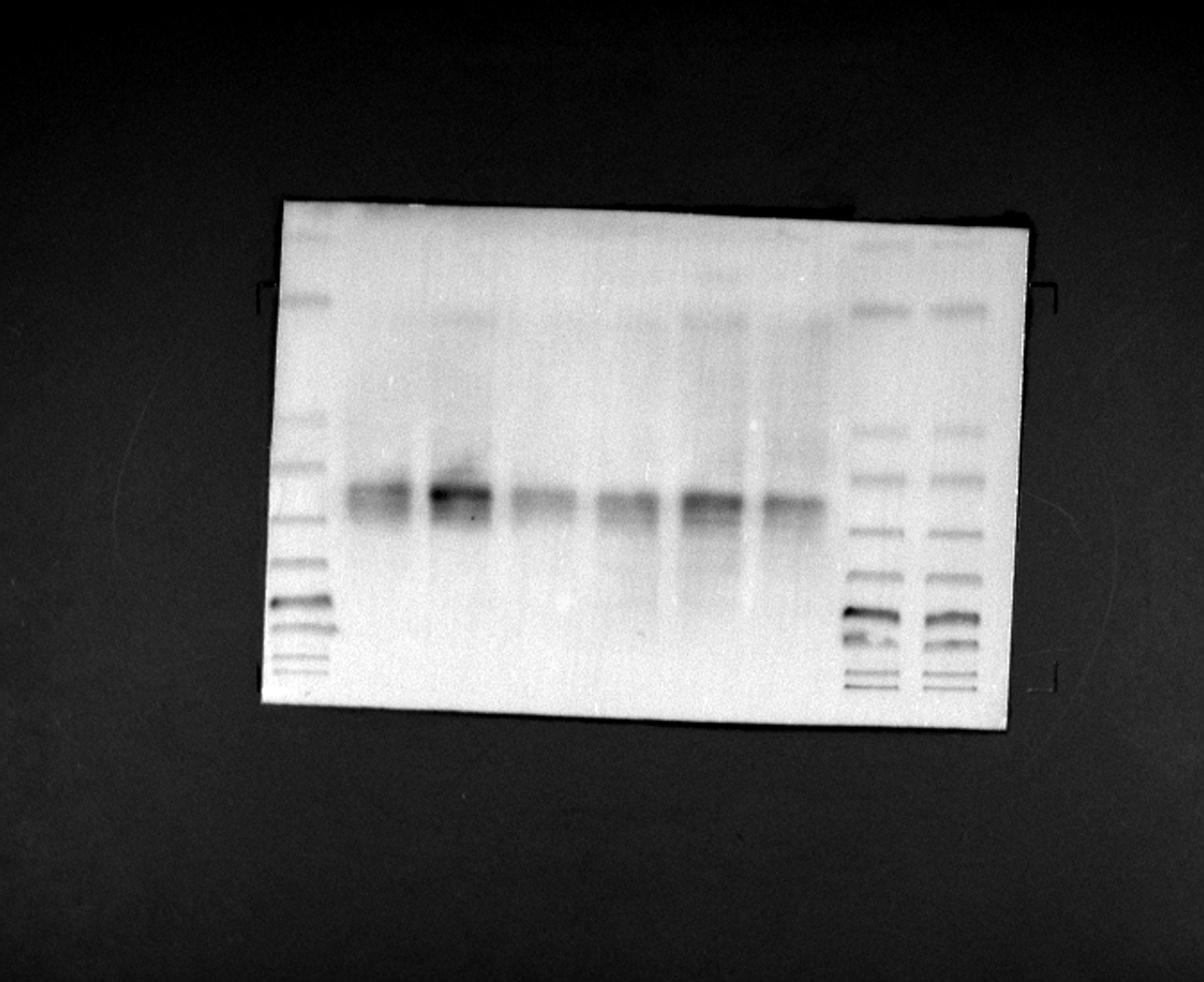

Supplement: Supplementary file 1 [file vetsci-12-00479-s001.zip › Western-blot/chicken/组织pikb-ikb-actin/ikb.Tif]

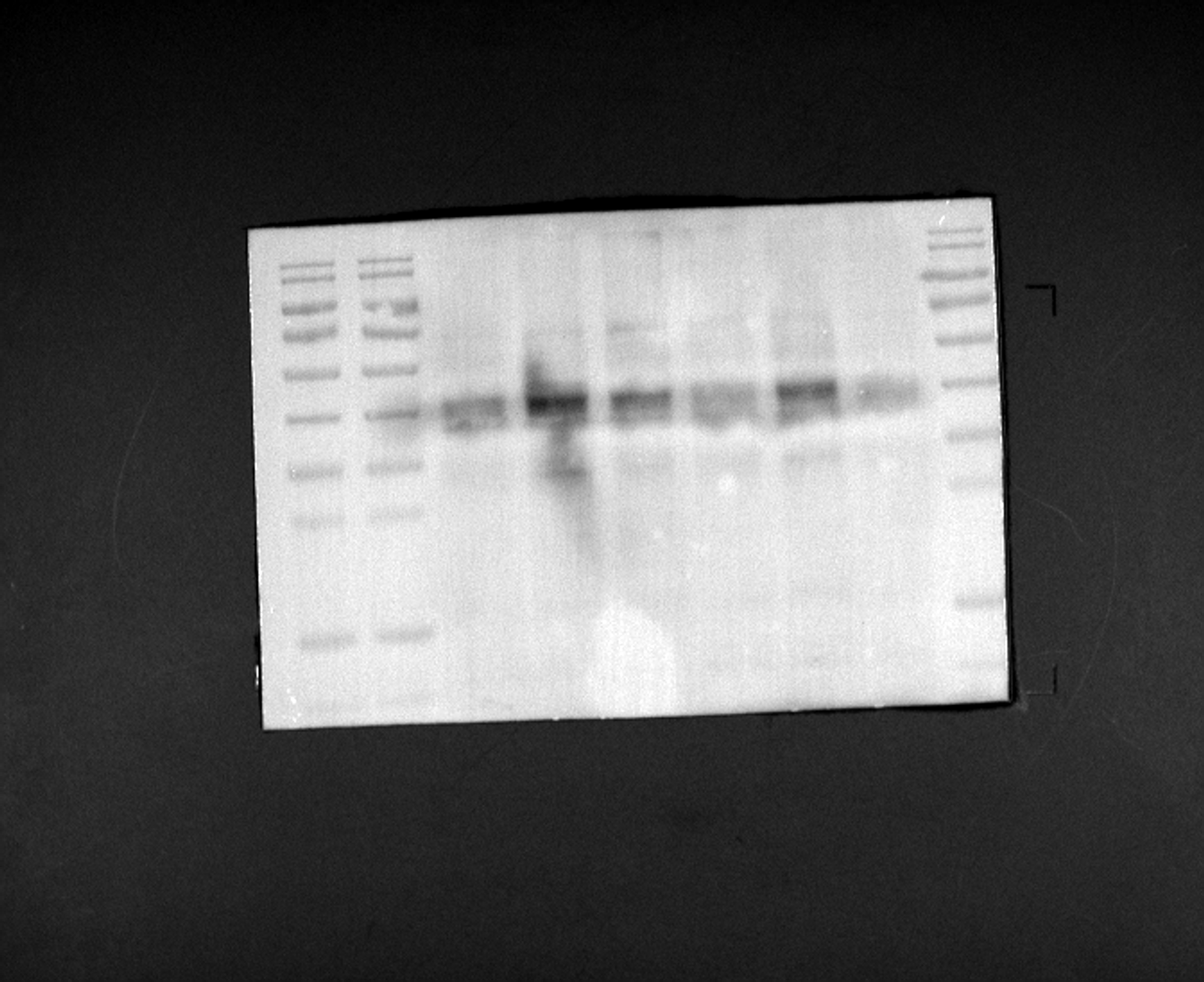

Supplement: Supplementary file 1 [file vetsci-12-00479-s001.zip › Western-blot/chicken/组织pikb-ikb-actin/PIKB.Tif]

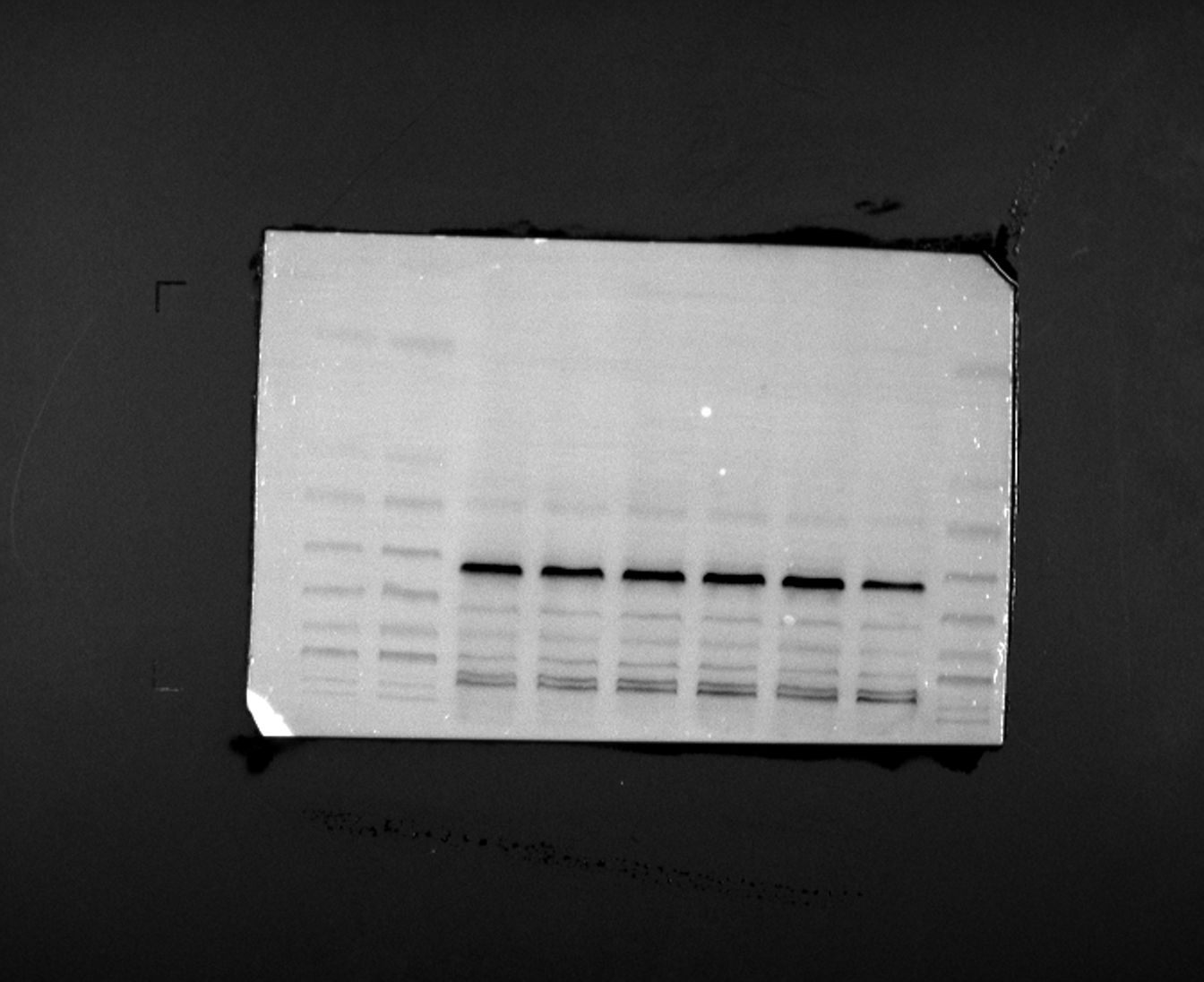

Supplement: Supplementary file 1 [file vetsci-12-00479-s001.zip › Western-blot/chicken/组织pp38-p38-actin/actin.Tif]

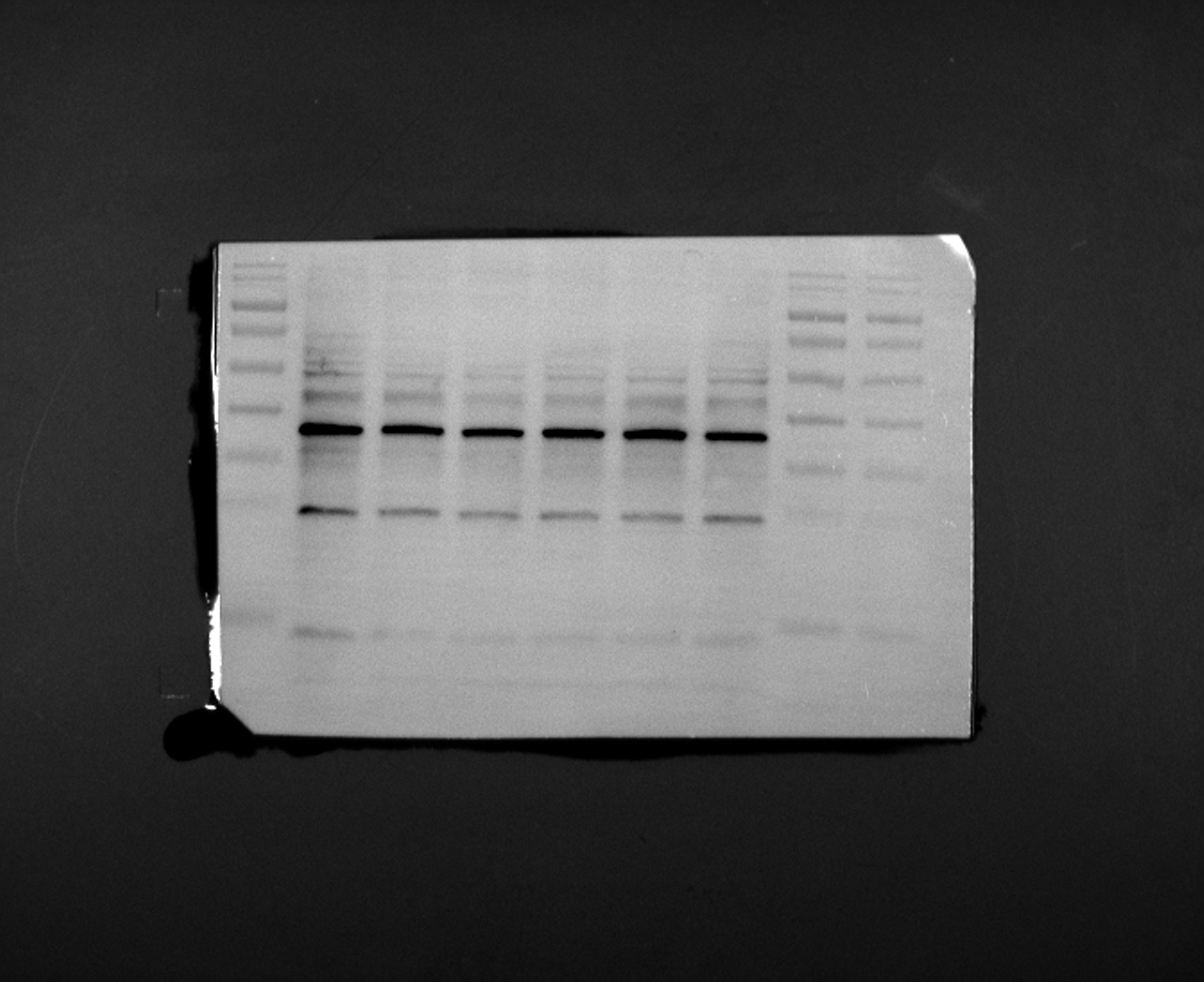

Supplement: Supplementary file 1 [file vetsci-12-00479-s001.zip › Western-blot/chicken/组织pp38-p38-actin/p38.Tif]

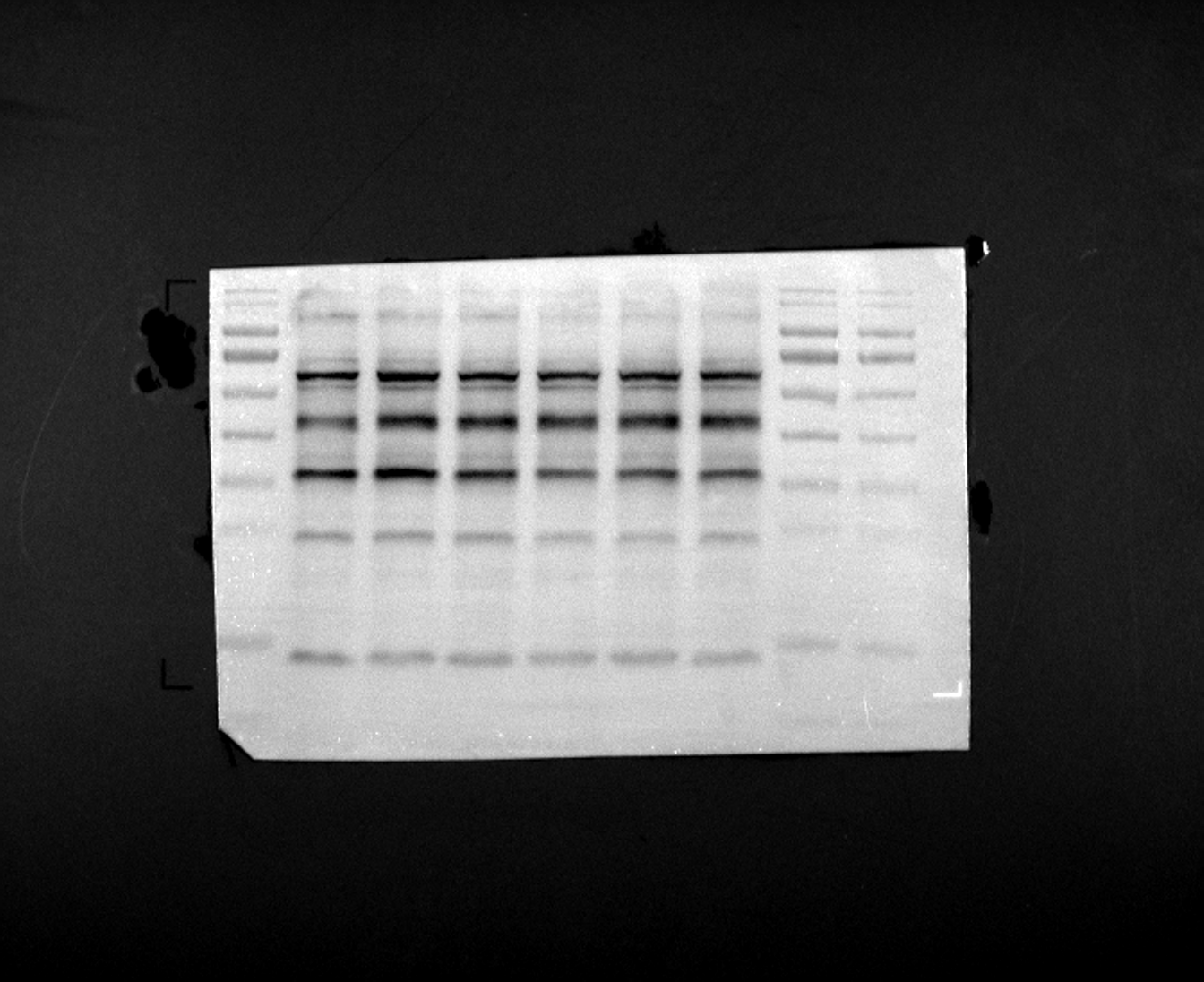

Supplement: Supplementary file 1 [file vetsci-12-00479-s001.zip › Western-blot/chicken/组织pp38-p38-actin/pp38.Tif]

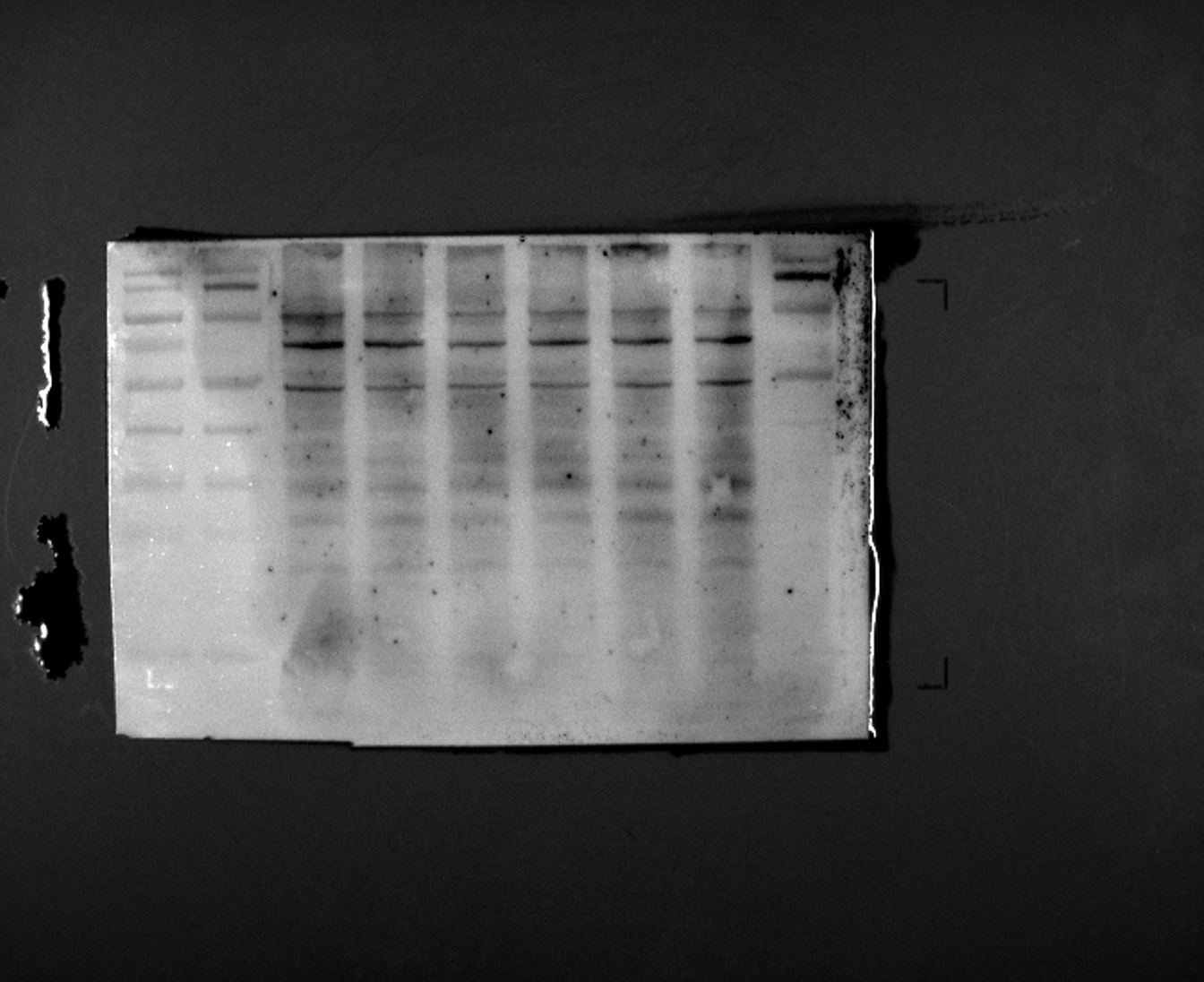

Supplement: Supplementary file 1 [file vetsci-12-00479-s001.zip › Western-blot/chicken/组织pp65-p65-actin/p65.Tif]

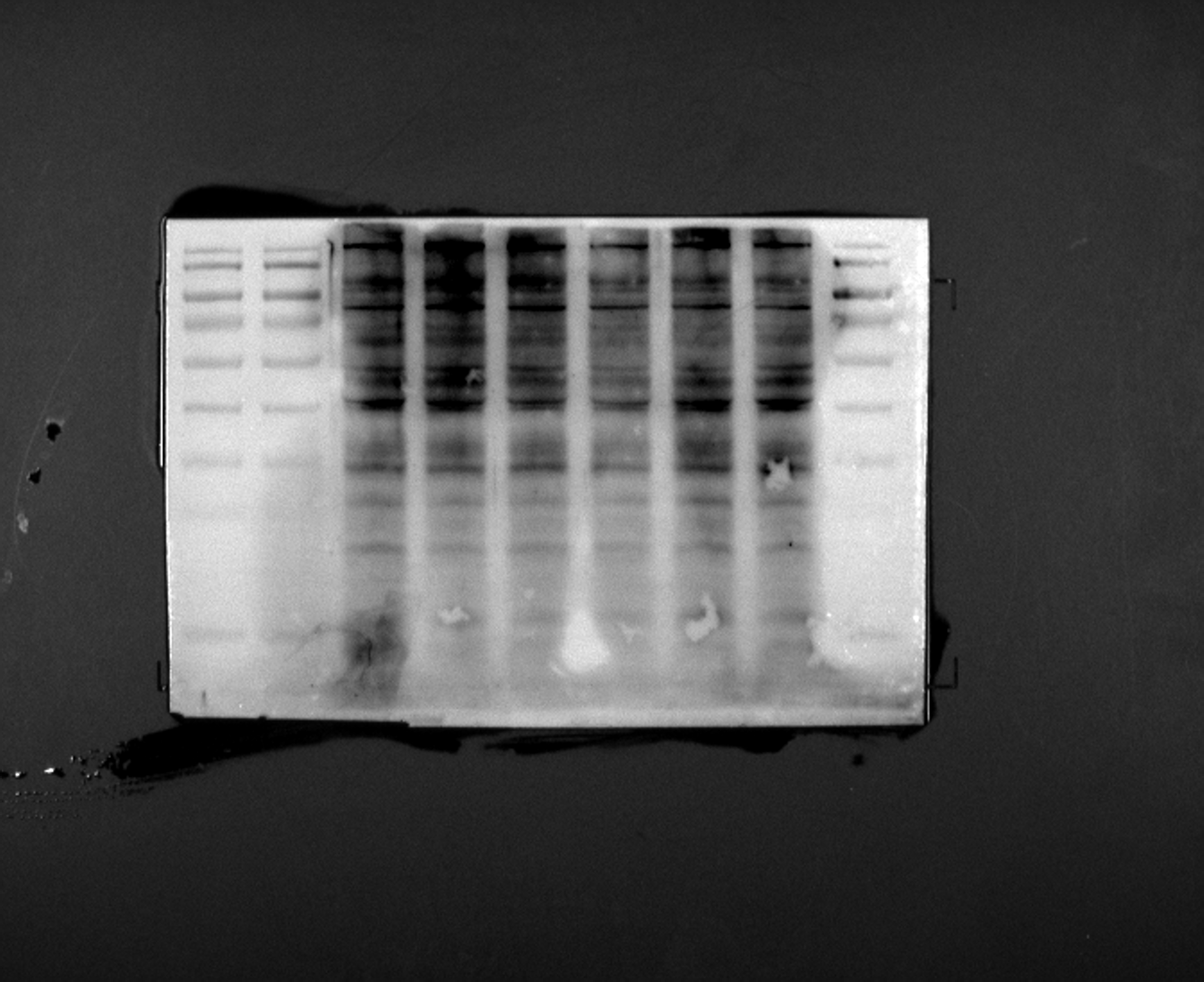

Supplement: Supplementary file 1 [file vetsci-12-00479-s001.zip › Western-blot/chicken/组织pp65-p65-actin/pp65.Tif]

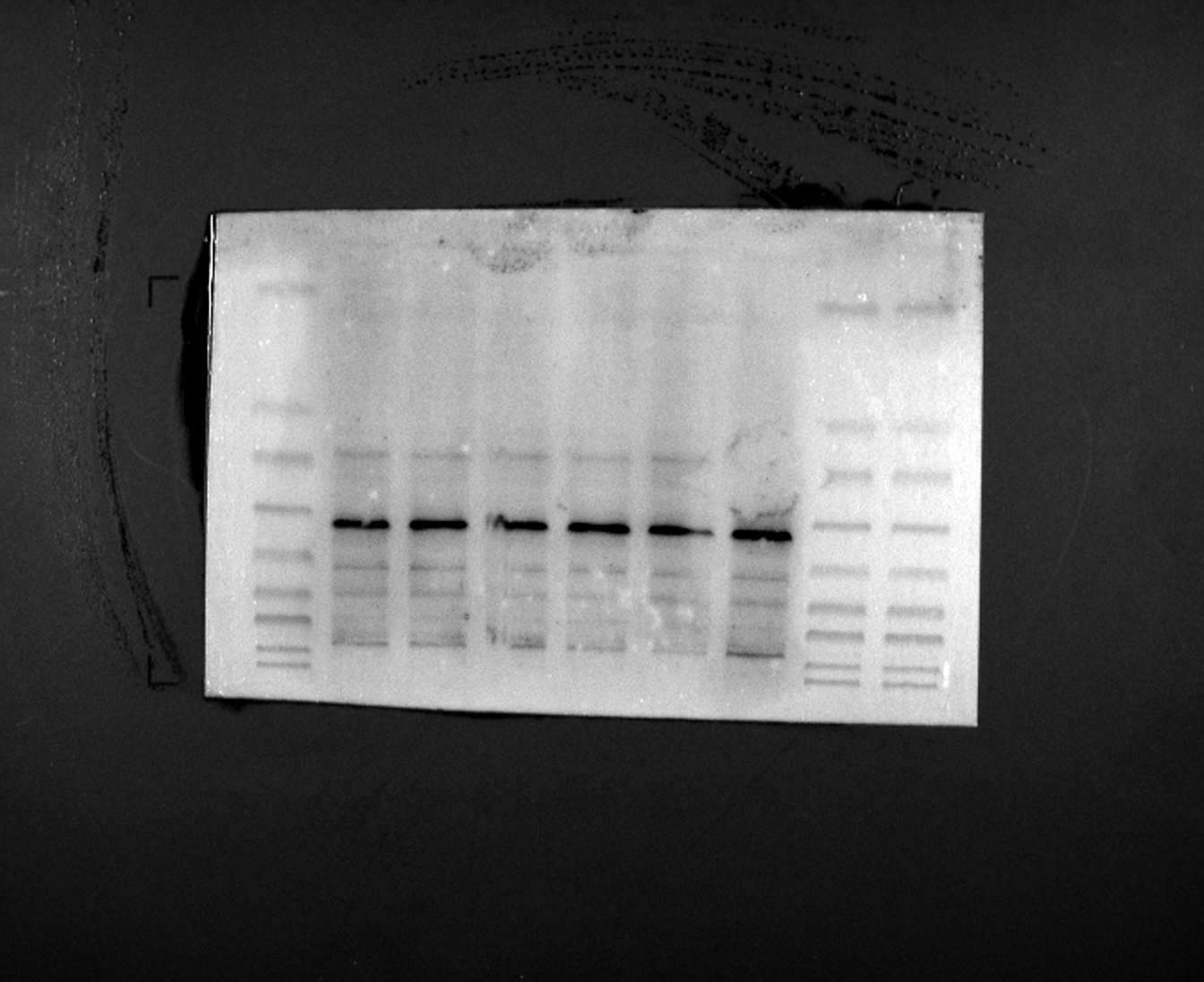

Supplement: Supplementary file 1 [file vetsci-12-00479-s001.zip › Western-blot/chicken/组织tlr4/actin.Tif]

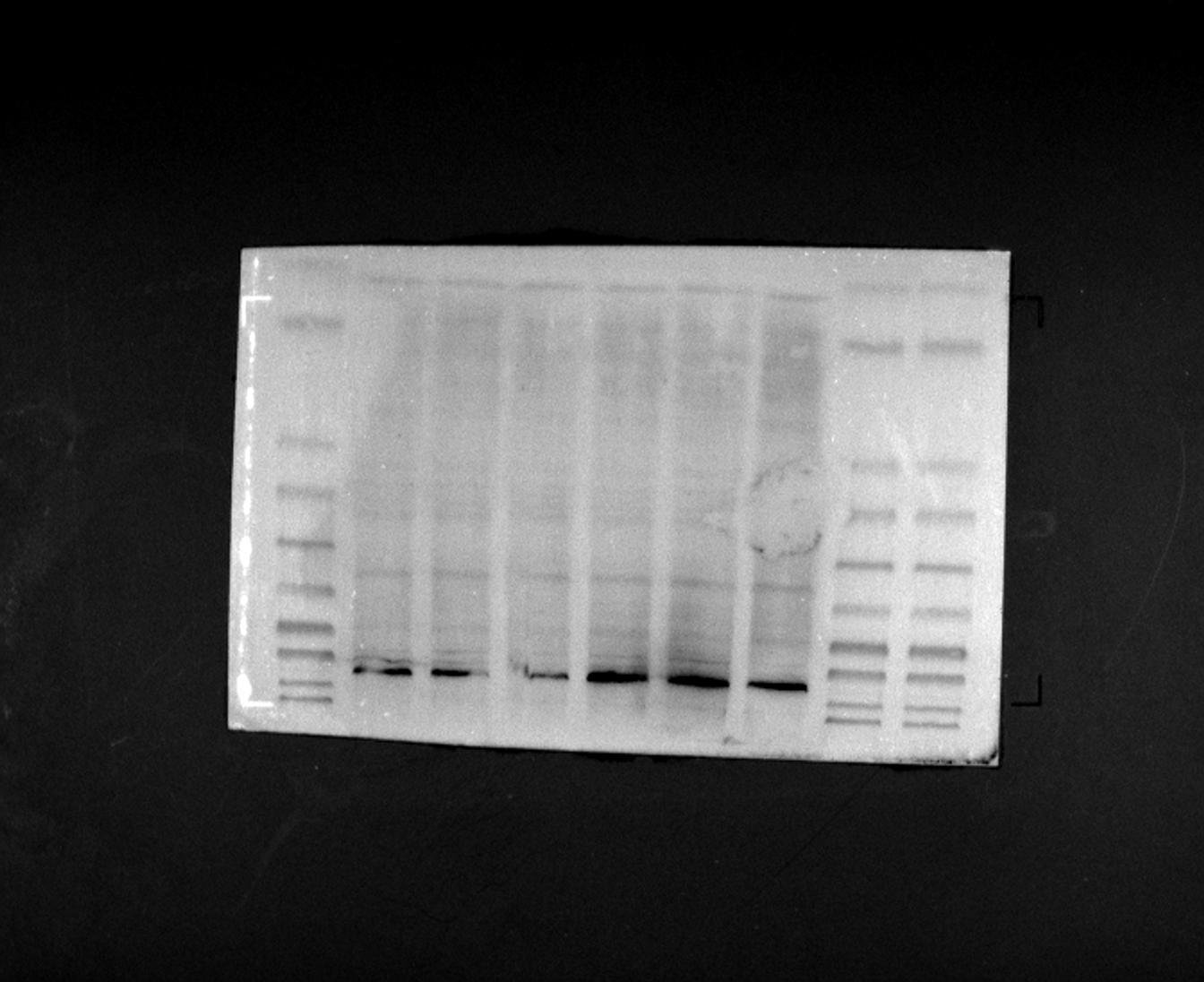

Supplement: Supplementary file 1 [file vetsci-12-00479-s001.zip › Western-blot/chicken/组织tlr4/tlr4.Tif]
